# KSHV encoded ORF59 modulates histone arginine methylation of the viral genome to promote viral reactivation

**DOI:** 10.1371/journal.ppat.1006482

**Published:** 2017-07-05

**Authors:** Roxanne C. Strahan, Maria McDowell-Sargent, Timsy Uppal, Pravinkumar Purushothaman, Subhash C. Verma

**Affiliations:** Department of Microbiology and Immunology, University of Nevada, Reno School of Medicine, Reno, NV, United States of America; University of Wisconsin-Madison, UNITED STATES

## Abstract

Kaposi’s sarcoma associated herpesvirus (KSHV) persists in a highly-ordered chromatin structure inside latently infected cells with the majority of the viral genome having repressive marks. However, upon reactivation the viral chromatin landscape changes into ‘open’ chromatin through the involvement of lysine demethylases and methyltransferases. Besides methylation of lysine residues of histone H3, arginine methylation of histone H4 plays an important role in controlling the compactness of the chromatin. Symmetric methylation of histone H4 at arginine 3 (H4R3me2s) negatively affects the methylation of histone H3 at lysine 4 (H3K4me3), an active epigenetic mark deposited on the viral chromatin during reactivation. We identified a novel binding partner to KSHV viral DNA processivity factor, ORF59-a protein arginine methyl transferase 5 (PRMT5). PRMT5 is an arginine methyltransferase that dimethylates arginine 3 (R3) of histone H4 in a symmetric manner, one hallmark of condensed chromatin. Our ChIP-seq data of symmetrically methylated H4 arginine 3 showed a significant decrease in H4R3me2s on the viral genome of reactivated cells as compared to the latent cells. Reduction in arginine methylation correlated with the binding of ORF59 on the viral chromatin and disruption of PRMT5 from its adapter protein, COPR5 (cooperator of PRMT5). Binding of PRMT5 through COPR5 is important for symmetric methylation of H4R3 and the expression of ORF59 competitively reduces the association of PRMT5 with COPR5, leading to a reduction in PRMT5 mediated arginine methylation. This ultimately resulted in a reduced level of symmetrically methylated H4R3 and increased levels of H3K4me3 marks, contributing to the formation of an open chromatin for transcription and DNA replication. Depletion of PRMT5 levels led to a decrease in symmetric methylation and increase in viral gene transcription confirming the role of PRMT5 in viral reactivation. In conclusion, ORF59 modulates histone-modifying enzymes to alter the chromatin structure during lytic reactivation.

## Author summary

Kaposi’s sarcoma-associated herpesvirus (KSHV) must carefully regulate both phases of its lifecycle in order to persist and proliferate effectively in the infected cells. In this study, we show the importance of dynamic epigenetic modifications on the viral chromatin in dictating whether KSHV displays the latent or lytic phase of its life cycle. Various chromatin-modifying enzymes are responsible for adding activating or repressive ‘marks’ on chromatin, one of these is a PRMT5 (protein arginine methyltransferase 5), which symmetrically dimethylates arginine 3 of histone H4 (H4R3me2s) and associates with condensed chromatin leading to restricted gene expression. An early lytic protein of KSHV, ORF59 associates with PRMT5 to disrupt its binding with the chromatin leading to a loss of repressive, H4R3me2s mark and corresponding gain of activating H3K4me3 during lytic reactivation.

## Introduction

Kaposi’s sarcoma-associated herpesvirus (KSHV), also known as human herpesvirus 8 (HHV8), is a member of the gammaherpesvirus family that is associated with Kaposi’s sarcoma (KS), Primary Effusion Lymphoma, a subset of Multicentric Castleman’s Disease, and (in HIV-co-infected patients) KSHV Inflammatory Cytokine Syndrome [1–4]. KSHV is a double-stranded DNA virus with a large genome that encodes for over 87 open reading frames (ORFs) including genes necessary for capsid, tegument, envelope, DNA replication and regulatory proteins. KSHV undergoes a bi-phasic lifecycle, common to other herpesviruses, that features both latent and lytic modes of infection. The virus persists indefinitely in the infected host in a latent form during which time only a small fraction of regulatory viral proteins are expressed, most notably the latency-associated nuclear antigen protein [5–7]. In the latent stage, LANA regulates latent genome replication and tethers the circular viral episomes to the host chromosomes to ensure the segregation of KSHV episomes to daughter cells upon cell division [8–11] Additionally, LANA modulates several signaling pathways to suppress the host immune antiviral responses to induce cell growth and survival [12–17].

During latency, the KSHV genome is maintained primarily in a heterochromatic conformation in which the genome is highly compact with restricted transcription of the viral genes [18, 19]. Specific ‘repressive’ epigenetic marks on the viral heterochromatin that contribute to the stability and tight regulation of gene expression include trimethylation of lysines 9 (H3K9me3) and 27 (H3K27me3) on histone H3, ubiquitination of lysine 119 of histone 2A (H2AK119Ub), and CpG-methylation [20]. The compactness of KSHV chromatin during latency was confirmed by sequencing the nucleosomal depleted DNA in FAIRE (Formaldehyde-Assisted Isolation of Regulatory Elements) assays, which revealed that only a small percentage of the viral genome, primarily the latency-associated regions, were in an active chromatin (euchromatin) state [18, 21, 22].

Latent viral genomes reactivate upon transcription of viral genes in a synchronized cascade of immediate early (IE), early (E), and late (L) genes, which leads to the production of infectious virion particles. Control of lytic reactivation is governed by the presence of both activating and repressive marks on the viral chromatin [19, 23, 24]. These are particularly important for certain regulatory regions of the KSHV genome with a bivalent chromatin structure because the balance between repressive and activating marks can tilt the scale for latency or reactivation [25, 26]. For example, the promoter of immediate early gene, RTA exists in a bivalent chromatin structure as it simultaneously possesses both activating, H3K4me3 and repressive, H3K27me3 marks [22]. Balance between these epigenetic marks is determined by multiple host cellular and viral factors [19–21, 25].

There is a growing list of epigenetic marks important for regulating chromatin structure and gene regulation [27]. One of the less studied epigenetic marks is arginine methylation, which is carried out by either type I or type II protein arginine methyltransferases (PRMTs) [28, 29]. PRMT5 is a type II methyltransferase that symmetrically dimethylates arginine 3 of histone H4, H4R3me2s [30–32]. PRMT5 mediated symmetric dimethylation of H4R3 promotes tri-methylation of histone, H3 lysine 27 (H3K27me3), a repressive mark that is one hallmark of compact chromatin [33]. In addition, knockdown of a related type II PRMT (PRMT7) is associated with a reduction in symmetric methylation of H4R3; moreover, reduced H4R3me2s levels corresponded to an increase in the activating, H3K4me3 marks [34]. The catalytic domain for the methyltransferase activity of PRMT5 was identified to be in Motif I (GAGRGP), and is essential for symmetrically methylating arginine 3 of histone H4 [31, 35]. PRMT5 has multiple versatile roles in cell growth and development and its association with specific binding partners influences substrate specificity and subcellular localization [29]. In the cytoplasm, pICln associates with PRMT5 and facilitates the methylation of Sm proteins, which increases their affinity for the SMN (survival of motor neuron) protein and facilitates proper assembly of the spliceosome for the formation of snRNPs [36, 37]. Another study of co-factors influencing substrate specificity of PRMT5 by Guderian et al. showed that PRMT5 bound to pICln or RioK1 recruits and symmetrically methylates nucleolin (a RNA binding protein) [38]. One of the cofactors important for regulating the histone methylation specificity of PRMT5 is MEP50 (also known as Wdr77) [39]. In addition to MEP50, another co-factor that regulates specific methylation of H4R3 by PRMT5 is a cooperator of PRMT5 (COPR5), which was identified in a yeast two-hybrid assay [40]. Recruitment of PRMT5 through COPR5 leads to a preferential symmetric dimethylation of histone H4R3 [40]. Importantly, symmetric dimethylation of H4R3 at the chromatin is associated with a compact chromatin causing transcriptional silencing [30, 41].

ORF59 is an early viral protein expressed within the first 24h of viral reactivation and acts as a processivity factor for the viral DNA polymerase during lytic DNA replication [42–44]. ORF59 homologs from other herpesviruses including human cytomegalovirus (hCMV) and Epstein-Barr virus (EBV) ppUL44 and BMRF1, respectively, are shown to be essential for lytic DNA replication [45]. The processivity factor dimerizes in the cytoplasm, binds to the viral DNA polymerase, and translocates it into the nucleus to assemble at the origin of lytic DNA replication (*OriLyt*) [46–49]. We recently reported that the phosphorylation of Ser378 and Ser379 is critical for ORF59’s activity, viral DNA synthesis, and virion production [50]. Although ORF59 was classically viewed as a processivity factor, studies have identified additional functions for ORF59; for example, ORF59 hinders the non-homologous end joining (NHEJ) repair of DNA double-stranded breaks by blocking the interaction between DNA-PKcs and the Ku complex during lytic replication to promote tumorigenesis [51]. Another report showed that ORF59 interacts with poly(ADP-ribose) polymerase 1 (PARP-1) and stimulates its proteosomal degradation to block the PARP-1 mediated cell cycle control and apoptosis [52].

In this study, we identified ORF59 binding proteins by immunoprecipitating the Flag epitope tagged ORF59 in BAC16 and classified bound proteins by mass spectroscopic analysis. The assay identified few viral proteins and a large number of cellular counterparts including RNA processing proteins and chromatin modifying enzymes. Here, we determined the association and significance of PRMT5 on viral chromatin and our data show that PRMT5 depletion in latent cells can trigger the transcription of lytic genes, suggesting PRMT5’s role in altering the chromatin landscape. Depletion of PRMT5 was linked to a reduced level of symmetric dimethylation of H4R3 (repressive mark) on viral chromatin, which correlated with lower H4R3me2s amounts detected during viral reactivation. Decrease in symmetric methylation during reactivation was linked to decreased levels of PRMT5 bound to the viral chromatin that facilitated the formation of euchromatin for active gene transcription. Our results confirmed that ORF59 competitively removed PRMT5 from its linker molecule, COPR5, which alters its specificity to symmetrically methylate H4R3. Furthermore, the loss of H4R3me2s marks lead to an increase in the tri-methylation of H3K4 (H3K4me3), an activating mark, and confirmed the role of symmetric methylation in regulating chromatin landscape. Taken together, we propose a mechanism by which ORF59 disrupts PRMT5’s mediated compact chromatin leading to the formation of open chromatin important for lytic replication.

## Results

**Fig 1:**
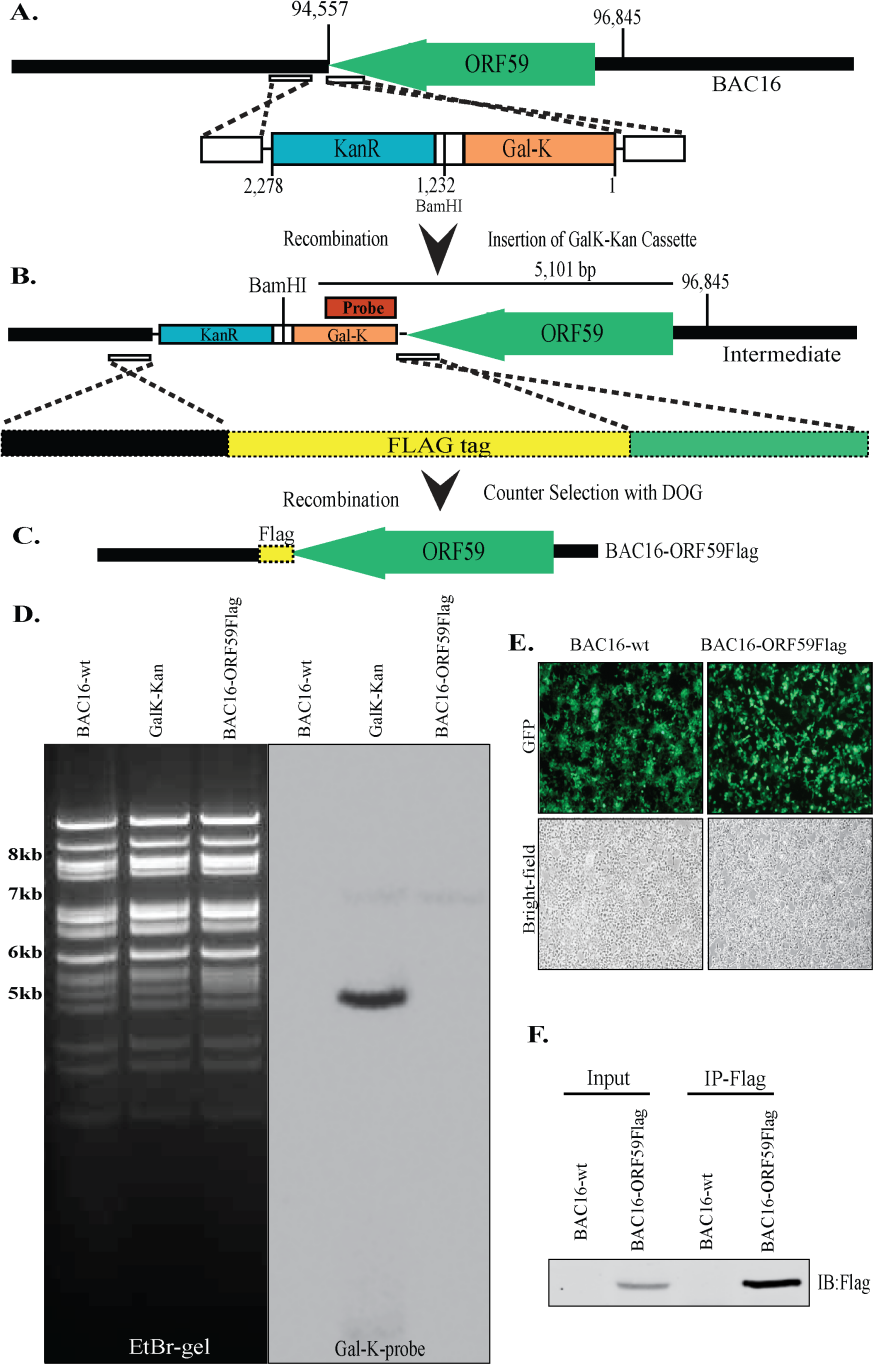
Schematic illustrating the flag epitope tagged ORF59 BACmid (BAC16-ORF59Flag) generation via homologous recombination. **A-C.** The flag epitope tag was inserted at the C-terminus of ORF59 by recombineering and GalK-KanR counter selection. Presence of GalK-Kan cassette was confirmed by BamHI digestion and Southern hybridization with GalK probe indicated in red (probe). Sequencing confirmed the insertion of Flag epitope tag. **D.** EtBr gel of BamHI digested BAC16-wt (lane 1), Intermediate containing GalK-KanR cassette (lane 2) and BAC16-ORF59Flag (lane 3). Southern blot with GalK probe showed expected 5,101bp band in the intermediate (lane 2). **E.** The HEK293L cells harbored stable BAC16-wt and BAC16-ORF59Flag. Green panels show the expression of GFP confirming the presence of Bac16WT or Bac16-ORF59Flag and the gray panels are the corresponding bright-field images. **F.** Flag tagged ORF59 was confirmed in the cell lysate (input lane) and by α–Flag immunoprecipitation and western blot (IP-Flag lane).

ORF59 encoded viral processivity factor (PF-8) assists viral DNA polymerase with DNA processivity during lytic DNA replication [47–49]. ORF59, an early protein of viral reactivation has also been shown to be involved in additional functions and the mutational studies determined ORF59 to be important for DNA replication and virion production [50–52]. Therefore, we were interested in identifying proteins associating with ORF59, which we achieved by generating a BACmid harboring Flag-epitope tagged ORF59 and immunoprecipitating ORF59 with anti-Flag antibody from BAC16-ORF59Flag 293L cells induced for 48h for lytic reactivation (Fig 1). Flag epitope tag was introduced at the C-terminus of ORF59 by homologous recombination (Fig 1A, 1B and 1C). BAC16 WT, intermediate containing *Gal*K-*Kan-R* and the final clone with Flag ORF59 was digested with BamHI and hybridized with *Gal*K probe to confirm the loss of GalK-KanR cassette after the negative selection (Fig 1D). These BACs, BAC16WT and BAC16-ORF59-Flag, were transfected into 293L cells to obtain clones maintaining viral genome (Fig 1E). Expression of Flag tagged ORF59 in BAC16-ORFF59, but not in BAC16WT, was confirmed by a western blot and immunoprecipitation (Fig 1F). Since BAC16-WT did not have Flag tagged ORF59, it was used as a control for anti-Flag (ORF59) immunoprecipitation. Proteins immunoprecipitated with ORF59-Flag were identified by LC/MS analysis at Mitch Hitchcock Nevada Proteomics Center, University of Nevada, Reno. A list of ORF59-specific binding proteins is shown in Tables 1 and 2 for viral and cellular proteins, respectively. This confirmed the binding of previously reported proteins and many additional uncharacterized proteins including KSHV mRNA transcript accumulation protein, ORF57 (Table 1). Although there were several proteins of interest, we focused on determining the role of protein arginine methyltransferase 5 (PRMT5) during viral reactivation due to its role in regulation of chromatin architecture (Table 2). Furthermore, 293 L cells stably expressing GFP fused ORF59 also precipitated PRMT5 identified by protein sequencing (Fig. A in S1 Text), confirming specificity of their association independent of other viral factors. PRMT5 is an arginine methyl transferase that symmetrically dimethylates the arginine 3 of histone H4 (H4R3me2s) of core histones by binding to the chromatin through a linker, COPR5, which leads to the formation of a compact chromatin structure. Binding of ORF59 with PRMT5 led us to speculate that ORF59 may disrupt PRMT5 mediated symmetric methylation of H4R3 to alter the viral chromatin for transcription and replication.

**Table 1:** Viral ORF59 binding partners. LC/MS analysis from anti-Flag immunoprecipitations on lytically induced 293LBAC16WT or 293LBAC16-ORF59Flag cells identified ORF59 associating viral proteins.

Viral Protein	Accession Number	Peptides Isolated
ORF59	PAP_HHV8P	17
ORF36	ORF36_HHV8P	4
ORF9	DPOL_HHV8P	3
ORF64	LTP_HHV8P	3
vIRF1	VIRF1_HHV8P	2
ORF10	ORF10_HHV8P	2
ORF54	DUT_HHV8P	2
ORF57	ICP27_HHV8P	4
ORF8	GB_HHV8P	1
ORF63	UL37_HHV8P	1
ORF75	ORF75_HHV8P	1
K5	MIR2_HHV8P	1
ORF6	DNBI_HHV8P	1
K3	MIR1_HHV8P	1
ORF62	VP19_HHV8P	1
ORF56	PRIM_HHV8P	1
ORF33	UL16_HHV8P	1
ORF27	ORF27_HHV8P	1
ORF24	ORF24_HHV8P	1
ORF18	UL79_HHV8P	1
ORF68	UL32_HHV8P	1
ORF69	UL31_HHV8P	1

**Table 2:** Cellular ORF59 binding partners. LC/MS analysis from anti-Flag immunoprecipitations on lytically induced 293LBAC16WT or 293LBAC16-ORF59Flag cells identified several ORF59 associating proteins. The top 100 binding partners with the highest affinity for ORF59 are shown in this table.

Cellular Protein	Accession Number	Peptides Isolated
DNA polymerase processivity factor	sp|F5HID2|PAP_HHV8P	58
Epididymis luminal protein 102	tr|B2ZZ89|B2ZZ89_HUMAN	9
Poly (ADP-ribose) polymerase 1	sp|P09874|PARP1_HUMAN	7
Isoform 2 of Heterogeneous nuclear ribonucleoprotein M	sp|P52272-2|HNRPM_HUMAN	8
Heat shock protein HSP90-beta	sp|P08238|HS90B_HUMAN	12
Leucine zipper protein 1	sp|Q86V48|LUZP1_HUMAN	5
Protein flightless-1 homolog	sp|Q13045|FLII_HUMAN	11
Elongation factor 2	sp|P13639|EF2_HUMAN	11
Protein arginine N-methyltransferase 5	sp|O14744|ANM5_HUMAN	7
X-ray repair complementing defective repair in chinese hamster cells 6 (Ku autoantigen) isoform CRA_a	tr|A0A024R1N4|A0A024R1N4_HUMAN	8
Histone H4	tr|B2R4R0|B2R4R0_HUMAN	3
ATP-dependent RNA helicase A	sp|Q08211|DHX9_HUMAN	8
cDNA FLJ78244, highly similar to homo sapiens eukaryotic translation initiation fator 4A, isoform 1 (EIF4A1)	tr|A8K7F6|A8K7F6_HUMAN	8
Isoform 2 of Dedicator of cytokinesis protein 7	sp|Q96N67-2|DOCK7_HUMAN (+1)	10
X-ray repair cross-complementing protein 5	sp|P13010|XRCC5_HUMAN	5
Pyruvate kinase	tr|V9HWB8|V9HWB8_HUMAN	8
Heterogeneous nuclear ribonucleoprotein U	sp|Q00839|HNRPU_HUMAN	7
Heterogeneous nuclear ribonucleoproteins A2/B1	sp|P22626|ROA2_HUMAN	4
Isoform 3 of Core histome macro-H2A.1	sp|O75367-3|H2AY_HUMAN (+1)	3
Non-POU domain-containing octamer-binding protein	sp|Q15233|NONO_HUMAN (+1)	4
Peroxiredoxin-1	sp|Q06830|PRDX1_HUMAN	4
Lamin-B1	sp|P20700|LMNB1_HUMAN	4
Fatty acid synthase	sp|P49327|FAS_HUMAN	7
Histone H2B type 1-J	sp|P06899|H2B1J_HUMAN	2
Probable ATP-dependent RNA helicase DDX17	tr|H3BLZ8|H3BLZ8_HUMAN (+1)	4
Splicing factor, proline—and glutamine—rich	sp|P23246|SFPQ_HUMAN	4
Nucleolar protein 56	sp|O00567|NOP56_HUMAN	3
60S ribosomal protein L	sp|P36578|RL4_HUMAN (+1)	4
ATP synthase subunit beta (fragment)	tr|Q0QEN7|Q0QEN7_HUMAN (+1)	6
Glyceraldehyde-3-phosphate dehydrogenase (GAPDH)	sp|P04406|G3P_HUMAN	2
Leucine-rich flightless-interacting protein 2	sp|Q9Y608|LRRF2_HUMAN (+1)	4
FACT complex subunit SPT16	sp|Q9Y5B9|SP16H_HUMAN	4
Annexin	tr|V9HW65|V9HW65_HUMAN	3
Guanine nucleotide-binding protein G(i) subunit alpha-2	sp|P04899|GNAI2_HUMAN	3
Alpha-enolase	sp|P06733|ENOA_HUMAN	4
RNA-binding protein 14	sp|Q96PK6|RBM14_HUMAN	2
Matrin-3	sp|P43243|MATR3_HUMAN (+1)	4
Bifunctional glutamate/proline-tRNA ligase	sp|P07814|SYEP_HUMAN	9
Insulin receptor substrate 4	sp|O14654|IRS4_HUMAN	5
Isoform 1A of Catenin delta-1	sp|O60716-5|CTND1_HUMAN	3
40S ribosomal protein S3	sp|P23396|RS3_HUMAN	3
Nucleolin	sp|P19338|NUCL_HUMAN	4
Nucleolar protein 58	sp|Q9Y2X3|NOP58_HUMAN	4
Isoform C1 of Heterogeneous nuclear ribonucleoproteins C1/C2	sp|P07910-2|HNRPC_HUMAN (+3)	3
Uveal autoantigen with coiled-coil domains and ankyrin repeats	sp|Q9BZF9|UACA_HUMAN	5
Inosine-5'-monophosphate dehydrogenase 2	sp|P12268|IMDH2_HUMAN	6
Ubiquitin-40S ribosomal protein S27a	sp|P62979|RS27A_HUMAN (+1)	4
40S ribosomal protein S3a	sp|P61247|RS3A_HUMAN (+1)	3
Isoform 3 of Exportin-2	sp|P55060-3|XPO2_HUMAN (+1)	2
Epididymis secretory protein Li 85	tr|Q53SS8|Q53SS8_HUMAN	1
C-1-tetrahydrofolate synthase, cytoplasmic	tr|F5H2F4|F5H2F4_HUMAN	7
NUMA1 variant protein (Fragment)	tr|Q4LE64|Q4LE64_HUMAN	2
Regulator of chromosome condensation 2, isoform CRA_a	tr|A0A024RAC5|A0A024RAC5_HUMAN	2
Isoform 4 of Heat shock protein 105kDA	sp|Q92598-4|HS105_HUMAN	6
Serine/arginine-rich splicing factor 1	sp|Q07955|SRSF1_HUMAN (+1)	1
Probable ATP-dependent RNA helicase DDX5	tr|J3KTA4|J3KTA4_HUMAN	5
14-3-3 protein theta	sp|P27348|1433T_HUMAN	6
Ubiquitin-activating enzyme E1 (A1S9T and BN75 temperature sensitivity complementing), isoform CRA_a	tr|A0A024R1A3|A0A024R1A3_HUMAN	6
Emerin	sp|P50402|EMD_HUMAN	1
Heterogeneous nuclear ribonucleoprotein F, isoform CRA_a	tr|A0A024R7T3|A0A024R7T3_HUMAN	3
Tyrosine-protein kinase Lyn	sp|P07948|LYN_HUMAN	2
HEAT repeat containing 1	tr|B2RWN5|B2RWN5_HUMAN	3
pre-rRNA processing protein FTSJ3	sp|Q8IY81|SPB1_HUMAN	2
Isoform 3 of Bcl-2 associated transcription factor 1	sp|Q9NYF8-3|BCLF1_HUMAN (+1)	4
Plasminogen activator inhibitor 1 RNA-binding protein	sp|Q8NC51|PAIRB_HUMAN	1
Leucine-rich PPR-motif containing protein	tr|E5KNY5|E5KNY5_HUMAN	4
Ribosomal protein L5, isoform CRA_b	tr|B3KTM6|B3KTM6_HUMAN	3
PC4 and SFRS1-interacting protein	sp|O75475|PSIP1_HUMAN	1
Helicase-like transcription factor	sp|Q14527|HLTF_HUMAN (+2)	3
EF-hand domain-containing protein D2	sp|Q96C19|EFHD2_HUMAN	4
RuvB-like 2	sp|Q9Y230|RUVB2_HUMAN	5
Transformer-2 protein homolog	sp|P62995|TRA2B_HUMAN (+3)	2
Isoform 3 of General transcription factor II-I	sp|P78347-3|GTF2I_HUMAN (+4)	5
Pre-mRNA-processing factor 19	sp|Q9UMS4|PRP19_HUMAN	3
Isoform 4 of Heterogeneous nuclear ribonucleoprotein Q	sp|O60506-4|HNRPQ_HUMAN	3
Isoform 5 of Double-stranded RNA-specific adensodine deaminase	sp|P55265-5|DSRAD_HUMAN	2
Apoptotic chromatin dondensation inducer in the nucleus	tr|E7EQT4|E7EQT4_HUMAN	2
Pre-mRNA-processing-splicing factor 8	sp|Q6P2Q9|PRP8_HUMAN	4
Zinc finger CCCH-type antiviral protein 1	sp|Q7Z2W4|ZCCHV_HUMAN	3
Peptidyl-prolyl cis-trans isomerase A	sp|P62937|PPIA_HUMAN (+1)	4
Guanine nucleotide binding protein-like 3 (Nucleolar), isoform CRA_b	tr|A0A024R2Z6|A0A024R2Z6_HUMAN	4
RuvB-like 1	sp|Q9Y265|RUVB1_HUMAN (+1)	4
tRNA-splicing ligase RtcB homolog	sp|Q9Y3I0|RTCB_HUMAN	3
Interleukin enhancer-binding factor 2	tr|B4DY09|B4DY09_HUMAN (+2)	2
DNA helicase	tr|B2RBA6|B2RBA6_HUMAN	3
Isoform 2 of DNA replication licensing factor MCM3	sp|P25205-2|MCM3_HUMAN	4
Isoform 3 of Double-stranded RNA-binding protein Staufen homolog 1	sp|O95793-3|STAU1_HUMAN	4
Eukaryotic translation initiation factor 5B	tr|A0A087WUT6|A0A087WUT6_HUMAN	3
High density lipoprotein binding protein (Vigilin), isoform CRA_a	tr|A0A024R4E5|A0A024R4E5_HUMAN (+1)	4
Staphylococcal nuclease domain-containing protein 1	sp|Q7KZF4|SND1_HUMAN (+2)	3
Endoplasmin (Heat Shock Protein 90kDa Beta Family Member 1)	sp|P14625|ENPL_HUMAN (+1)	2
Isoform 2 of tRNA (cytosine(34)-C(5))-methyltransferase	sp|Q08J23-2|NSUN2_HUMAN	2
ATP-binding cassette sub-family F member 2	sp|Q9UG63|ABCF2_HUMAN (+1)	2
40S ribosomal protein S6	tr|A2A3R6|A2A3R6_HUMAN (+1)	2
Proliferation-associated 2G4, 38kDa, isoform CTA	tr|A0A024RB85|A0A024RB85_HUMAN (+2)	1
60S ribosomal protein P2	sp|P05387|RLA2_HUMAN	1
N-acetyltransferase 10	sp|Q9H0A0|NAT10_HUMAN	4
Isoform 2 of ATP-binding cassette sub-family F member 1	sp|Q8NE71-2|ABCF1_HUMAN (+1)	4
Eukaryotic translation initiation factor 3 subunit A	sp|Q14152|EIF3A_HUMAN (+2)	4
Protein RRP5 homolog	sp|Q14690|RRP5_HUMAN	5

### ORF59 associates with PRMT5

**Fig 2:**
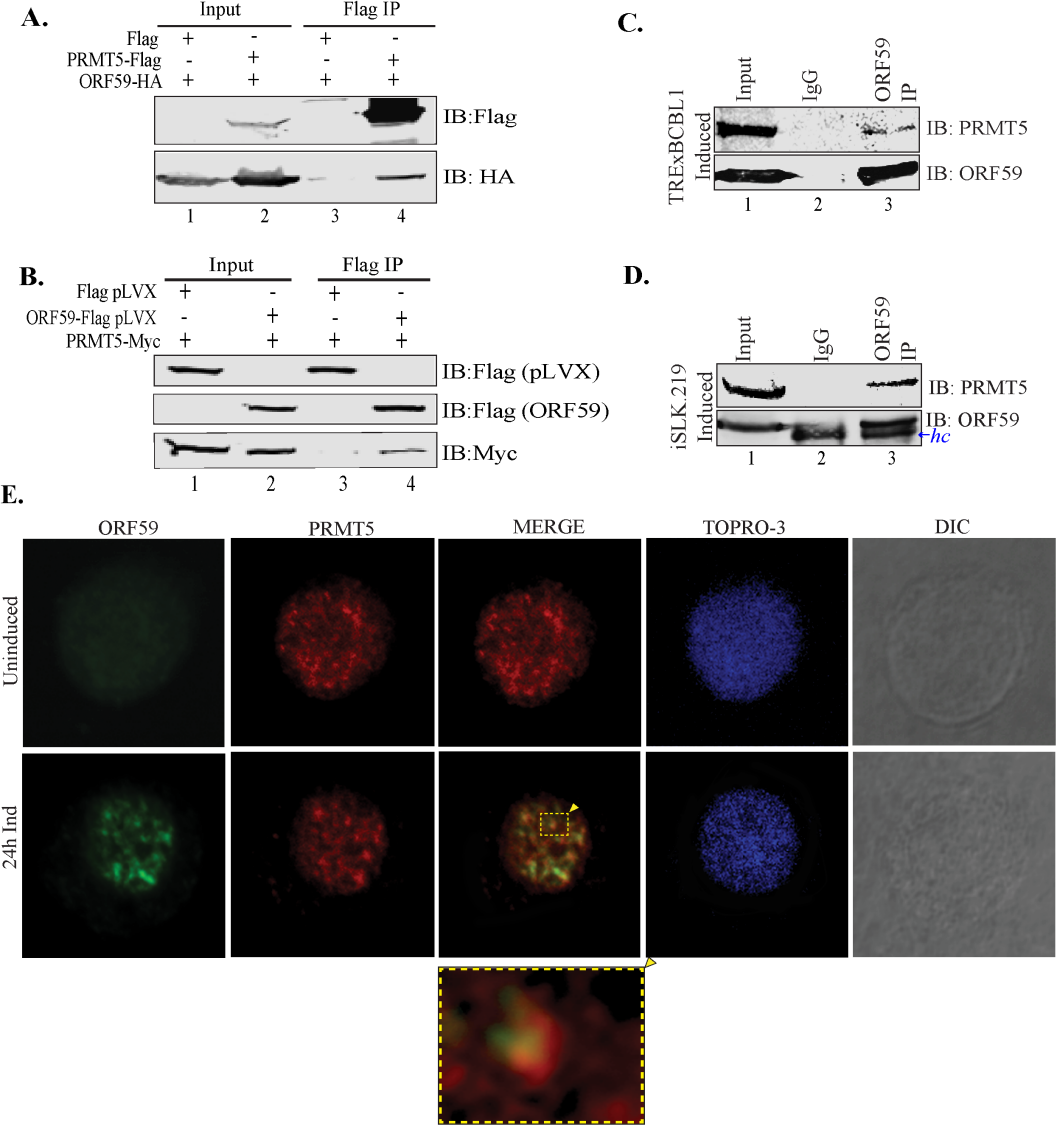
ORF59 associates with PRMT5. **A.** Binding between full-length ORF59 and PRMT5 was established by cotransfecting ORF59-HA and PRMT5-Flag plasmids and immunoprecipitating with anti-Flag antibody. The western blot shows specific binding between PRMT5 and ORF59 (lane 4), as the vector alone did not precipitate any ORF59 (lane 3). **B.** A reverse co-immunoprecipitation demonstrates binding between ORF59 and PRMT5. ORF59-Flag was cotransfected with PRMT5-Myc and the lysate was subjected to immunoprecipitation with anti-Flag antibody. These two proteins showed specific association as lane 4 shows PRMT5-Myc band but the vector alone did not precipitate any PRMT5-Myc. **C,** and **D.** KSHV-positive. TRExBCBL1-Rta cells or iSLK.219 cells were induced with doxycycline/NaB before harvesting for immunoprecipitation of ORF59. Lane 3 demonstrates a specific association between ORF59 and endogeneous PRMT5 (lane 3) while IgG control did not show detectable levels of PRMT5 (lane 2). Heavy chain-*hc*. **E.** Uninduced or 24h doxycycline induced TRExBCBL1-Rta cells were fixed on glass coverslips and stained for ORF59 and PRMT5 localization using specific antibodies. These cells were counter-stained with TO-PRO3 for the detection of nuclei. Images of showed colocalization of ORF59 and PRMT5 in the cell nucleus. Merge signals were magnified to demonstrate colocalization. iSLK.219 cells were not included in the IFA assay because of GFP backbone in the viral genome and express RFP after induction.

In order to confirm the binding of PRMT5 with ORF59, we performed co-immunopreciptation assays on endogenous as well as over expressed proteins. First, the ORF59-HA and PRMT5-Flag expression vectors were transfected into 293T cells and an immunoprecipitation with anti-Flag antibody for PRMT5 co-precipitated the HA-tagged ORF59 (Fig 2A, lane 4). Lack of a detectable band of ORF59-HA with control vector (Flag), confirmed that these proteins associate specifically (Fig 2A, lane 3). Furthermore, the reverse CoIP in which PRMT5-Myc with ORF59-Flag or Flag vector were expressed and immunopreciptiated with anti-flag antibody, showed specific precipitation of PRMT5-Myc (Fig 2B, lane 4). Flag Vector was unable to precipitate PRMT5-Myc (Fig 2B, lane 3), which confirmed specificity of the assays.

Next, we used KSHV-infected cell lines, TRExBCBL1-RTA and iSLK.219 to affirm this finding in an endogenous system. Each cell line was induced for lytic replication by treatment with doxycycline and ORF59 was immunoprecipitated with anti-ORF59 antibody. Immune detection of PRMT5 in ORF59 precipitated lanes showed a distinct band in both, TRExBCBL-1 and iSLK.219 cell lines and confirmed the association of these two proteins during lytic reactivation (Fig 2C and 2D, lanes 3). The control antibody, IgG did not co-precipitate any PRMT5, which again confirmed specificity of the assay (Fig 2C and 2D, lanes 2).

After we confirmed the binding between these two proteins, we were interested in determining whether they localize to the same nuclear compartment during viral reactivation. To test this, we performed immune localization of these proteins in doxycycline induced TRExBCBL1-RTA cells. These proteins were detected using respective antibodies. Both ORF59 and PRMT5 are nuclear proteins that showed nuclear localization, as expected, and many of these foci showed localization of both proteins in the same nuclear compartment (Fig 2E, merge panel). Latent (uninduced) cells do not express ORF59, therefore undetected, but the subcellular localization of PRMT5 was seen to be similar as in the induced cells (Fig 2E). This confirmed that ORF59 associates with PRMT5 in reactivated cells.

### ORF59 binds at the methyltransferase site of PRMT5

**Fig 3:**
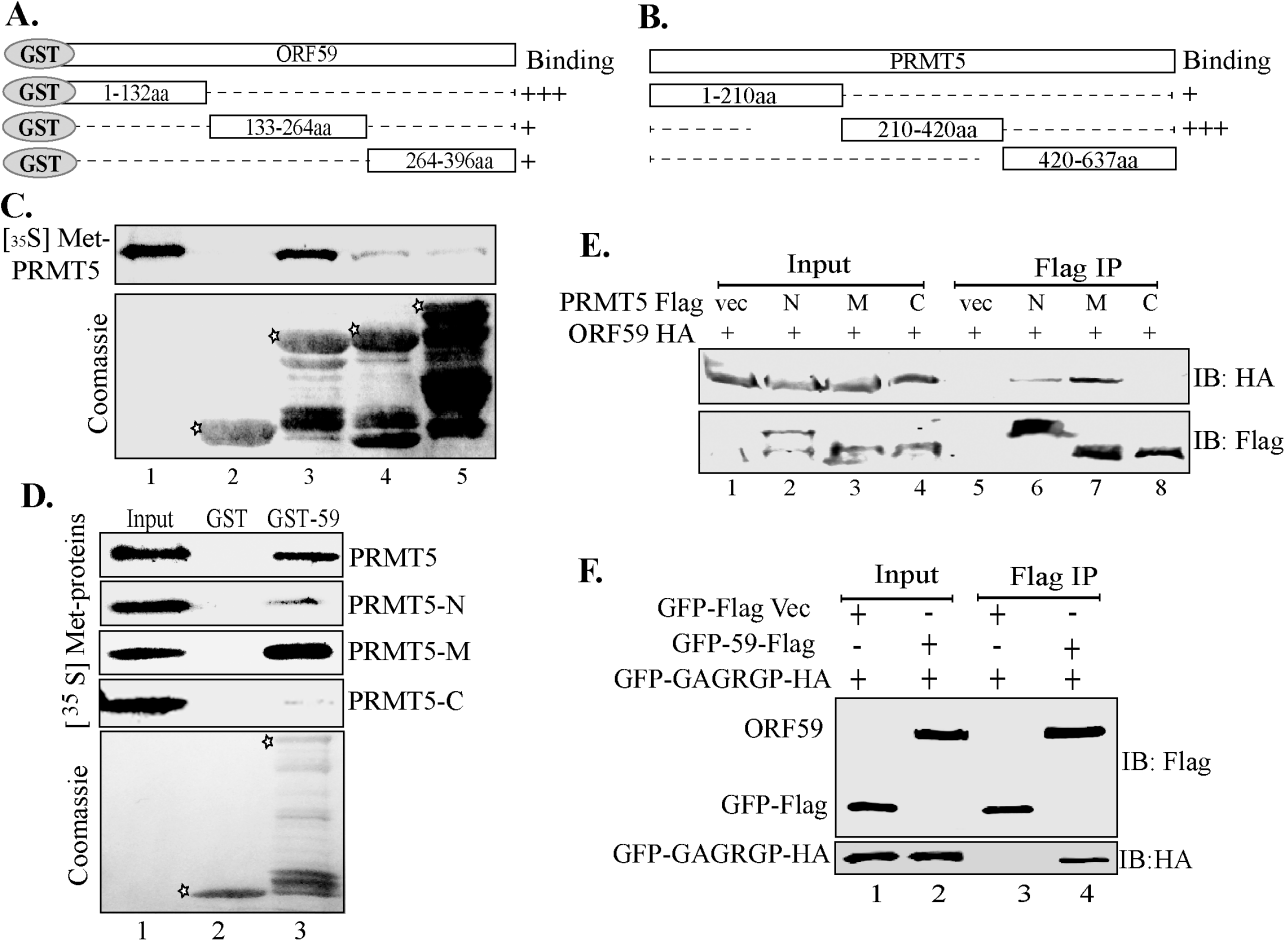
ORF59 binds to the middle (210-420aa) segment of PRMT5. **A.** Schematic of ORF59 and its truncations fused to GST. **B.** Domains of PRMT5 used in binding assays. **C.** *In vitro* translated ^35^S-methionine labeled-PRMT5 subjected to binding with truncated domains of ORF59 fused to GST. The N-terminal domain, 1-132aa of ORF59 bound most strongly with PRMT5 (lane 3), while control GST (lane 2) did not show any detectable band demonstrating specificity. Asterisks on the coomassie gel indicate GST fused segments of ORF59 used in the binding assay. **D.** *In vitro* translated ^35^S-methionine labeled PRMT5 full length and its truncation (amino-N, middle-M and carboxyl-C) mutants were subjected to binding with control GST (GST) (lane 2) or GST-59 (lane 3). PRMT5 residues 210-420aa (middle segment) associated most strongly with GST-ORF59 (lane 3). Asterisks indicate Control GST (lane 2) and full-length ORF59 fused GST (lane 3) in the coomassie image. **E.** Flag-tagged PRMT5 truncations (amino-N, middle-M and carboxyl-C) mutants were co-expressed with HA-epitope tagged ORF59 and lysates were immunoprecipitated with anti-Flag antibody. PRMT5 210-420aa immunoprecipitated ORF59 specifically as compared to Flag-vector (compare lanes 5 with 7) and PRMT5 1-210aa also showed a weaker association with ORF59 (lane 6). **F.** The middle domain of PRMT5 containing the catalytic domain (GAGRGP) was fused to GFP and HA and co-expressed with ORF59-Flag or vector control GFP-Flag and lysates were immunoprecipitated with anti-Flag antibody. ORF59 associated with the GFP-HA-tagged PRMT5 catalytic domain but vector control GFP alone did not (compare lane 4 with 3, IB: HA panel).

To further investigate the association between ORF59 and PRMT5, a systematic series of truncation constructs of these proteins were generated (Fig 3A and 3B). The association of ORF59 with PRMT5 was first tested by using GST fused ORF59 truncations with *in vitro* translated ^35^S-methionine labeled full-length PRMT5. GST-tagged ORF59 segments or GST-control were used to precipitate the *in vitro* translated PRMT5 (Fig 3C). The first segment of ORF59 (1-132aa) precipitated PRMT5 most strongly compared to the other segments indicating the segment to be the primary site of PRMT5’s binding (Fig 3C, compare lane 3 with lanes 4 and 5). The specificity of their binding was confirmed by the lack of any bound PRMT5 with GST control (Fig 3C, lane 2). The binding assay was conducted with equal amounts of *in vitro* translated PRMT5 represented in the input lane (Fig 3, lane1), while GST proteins used for binding are shown with coomassie staining (Fig 3C, bands marked with asterisks).

Next, we determined the domain of PRMT5 primarily responsible for its interaction with ORF59. Full-length ORF59 fused to GST (GST-59) or control GST was used to precipitate the *in vitro* translated PRMT5 and its truncations. Equal proportions of the *in vitro* translated PRMT5 and respective truncations were used in the binding assays with GST or ORF59-GST. As expected, ORF59-GST but not control GST precipitated full-length PRMT5 (Fig 3D. lane 3, PRMT5 panel). Interestingly, ORF59-GST bound most strongly with the PRMT5 210-420aa region (Fig 3D. lane 3, PRMT5-M panel). Although a small proportion of PRMT5 1-210aa also bound with ORF59-GST it is very evident that ORF59 interacts most robustly with the 210-420aa regions of PRMT5, which notably possesses the catalytic domain for methyltransferase activity.

This association was further confirmed using an over-expression system in which HEK293T cells were transfected with the various truncation mutants of PRMT5 (Flag epitope tagged) and with HA-tagged ORF59. Immunoprecipitations with anti-Flag antibody for PRMT5 truncations showed efficient binding of PRMT5 segment 210-420aa with ORF59 detected in a western blot with HA antibody (Fig 3E, lane 7). The binding of PRMT5 truncations with ORF59 showed similar pattern as detected with the *in vitro* translated proteins i.e. PRMT5-M (210-420aa) of PRMT5 associated most strongly with ORF59 (Fig 3E). The N-terminal domain of PRMT5 showed also showed binding, although lower compared to the middle region, which is similar to the *in vitro* binding data (Fig 3E, compare lane 6 and 7). The C-terminal domain of PRMT5 did not associate with ORF59 in both *in vitro* binding and immunoprecipitation assays (Fig 3D and 3E). Since the middle region of PRMT5 (PRMT5-M) was the primary site of interaction with ORF59, and possesses the catalytic domain required for methyltransferase activity, we wanted to determine whether the minimal catalytic domain associated with ORF59 [31]. To do so, we constructed a PRMT5 truncation containing the catalytic motif fused to GFP and HA. This was used for binding with GFP tagged ORF59-Flag or GFP-Flag (Fig 3F). Immunoprecipitation with anti-Flag antibody showed specific precipitation of catalytic domain of PRMT5 with GFP-59-Flag but not with the GFP-Flag control (Fig 3F). This confirmed that ORF59 associated with PRMT5 through its N-terminal domain (1-132aa) to the middle, catalytic region of PRMT5, possibly to interfere with the methyltransferase activity.

### PRMT5 depletion reduced H4R3me2s levels and upregulated viral gene transcription

**Fig 4:**
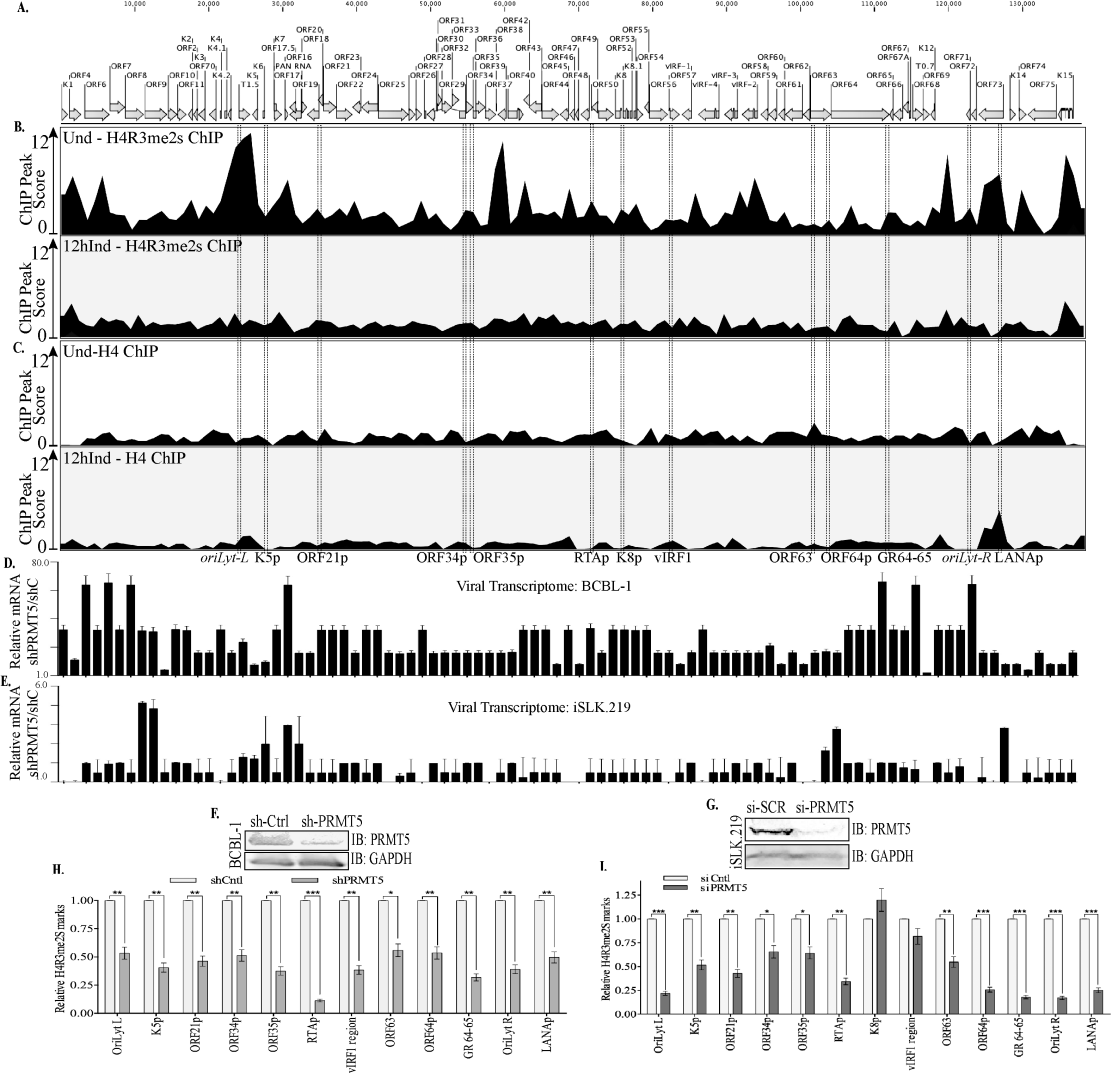
Symmetric di-methylation of H4R3 (H4R3me2s) levels were significantly reduced in lytic reactivated and PRMT5 depleted cells. **A.** Schematic of the KSHV genome with open reading frames indicated. **B.** Chromatin from uninduced TRExBCBL1-RTA cells was immunoprecipitated with anti-H4R3me2s antibody followed by next-generation sequencing of the ChIP DNA (ChIP-Seq) and detection of ChIP peaks. Chromatin from 12h doxycycline-induced TRExBCBL1-RTA cells was immunoprecipitated with anti-H4R3me2s antibody followed by next-generation sequencing of the ChIP DNA (ChIP-Seq) and detection of ChIP peaks. The peak score represents peak height. The prevalence of H4R3me2s marks was reduced on viral genome undergoing lytic reactivation. C. Histone H4 on the latent and 12h induced TRExBCBL1-RTA cells were determined by mapping the reads to the KSHV reference genome and then detecting ChIP peaks. **D.** Relative expression of viral genes in PRMT5 depleted (shPRMT5) TRExBCBL1-Rta cells as compared to the control (shCtrl) cells. Viral genes detected by qPCR showed an enhanced gene transcription in the absence of PRMT5 relative to the control. **E.** Relative expression of viral genes in PRMT5 depleted (siPRMT5) iSLK.219 cells as compared to the control, scrambled (si-Ctrl) cells. Transcriptome analysis of entire KSHV genome by qPCR showed an enhanced expression of many genes in PRMT5 depleted (si-PRMT5) as compared to the control (si-Ctrl) cells. **F.** BCBL-1 cells stably transduced with PRMT5 shRNA expressing lentiviruses showed depletion of PRMT5 levels as compared to the control shRNA transduced cells. **G.** iSLK.219 cells were transfected with si-Control or si-PRMT5 showed reduction of PRMT5 in si-PRMT5 cells. **H.** BCBL-1 cells depleted with PRMT5 in panel F were subjected for chromatin immunoprecipitation with anti-H4R3me2s antibody and analyzed by qPCR at selected region at promoters, which displayed differential levels of H4R3me2s in our ChIP-Seq results. PRMT5-depletion displayed significantly lower levels of H4R3me2s than shControl cells al all the tested regions. **I.** iSLK.219 cells depleted with PRMT5 from panel G were subjected ChIP assay with anti-H4R3me2s antibody and analyzed by qPCR at the same regions used on panel H showed almost similar reduction of H4R3me2s bound chromatin at those regions.

A balance between repressive and activating epigenetic marks on the KSHV genome has been shown to regulate the viral gene expression and control the switch between latent-lytic cycles of the virus [19–21, 25]. The immediate early gene, RTA promoter region is one example of bivalent chromatin, displaying both repressive, H3K27me3 and activating, H3K4me3 marks but upon reactivation activating marks are enriched following a decrease of the repressive marks. This is a particularly ingenious mechanism as it allows the virus to respond to environmental stimuli rapidly. PRMT5, an arginine methyltransferase responsible for the H4R3me2s modification, promotes heterochromatinization. To understand the importance of PRMT5 and the symmetric methylation of histone H4R3 during the viral life cycle, we determined the symmetric methylation levels on H4R3 of viral chromatin during latency and lytic reactivation. We immunoprecipitated symmetrically dimethylated H4R3 containing chromatin from un-induced and doxycycline induced TRExBCBL1-RTA cells. Specificity of the antibody for the symmetrically methylated form of H4R3 chromatin was confirmed by immunoprecipitation and detection with symmetric antibody, which did not cross-react with asymmetric form (Fig. B in S1 Text). DNA extracted from the bound chromatin was sequenced (ChIP-Seq) and analyzed for enriched regions (ChIP-peaks) using the ChIP analysis tool of CLC Workbench [53]. The peak score representing the relative levels of H4R3me2s containing chromatin was detected throughout the genome with enrichment at specific regions on the uninduced latent genome (Fig 4B). Interestingly, the levels of symmetrically methylated H4R3 (H4R3me2s) chromatin on the lytically reactivated genome were significantly reduced throughout the genome with certain regions totally devoid of the H4R3me2s chromatin (Fig 4B). Considering the transcriptionally restrictive nature of the latent genome in contrast to highly active viral gene transcription during reactivation, the abundance of symmetrically methylated H4R3 in latent cells and subsequent reduction in lytic cells was to be expected (Fig 4B). In addition, we tested histone H4 occupancy on viral genome by performing histone H4 ChIP-Seq on the same latent and lytic TRExBCBL1-RTA cells and analyzing for any enriched ChIP-peaks (Fig 4C). Mapping the reads of histone H4 ChIP-seq to the viral genome showed a consistent occupancy and the ChIP peak calling software did not detect peaks with significant peak core due to the uniformed presence of histone H4 throughout the genome. Importantly, the occupancy of histone H4 on the viral genome remained similar after induction suggesting that reduction in H4R3me2s during induction was not due to overall reduction of histone H4 from the viral genome. To further ensure that the changes in H4R3me2s enrichment were not due simply to changes in genome copy numbers, we tested the levels in TREx-BCBL1-RTA cells induced for lytic replication for 12h and treated with replication inhibitor, 0.5mM PFA. At 12h time point, we still detected significant reduction in H4R3me2s enrichment at indicated region of the viral genome including *OriLyt*, RTA promoter, K8 promoter, and ORF21 promoter (Fig. C in S1 Text).

Since PRMT5 symmetrically methylates H4R3 to make the chromatin compact, we wanted to determine whether depleting PRMT5 would be sufficient to alter the chromatin into a transcriptionally active form. To this end, we performed PRMT5 knockdown by transducing shRNA in KSHV positive, BCBL-1 cells and transfection of siRNA on iSLK.219 cells. Both shRNA and siRNA for PRMT5 significantly reduced the levels of PRMT5 compared to the shControl and scrambled siRNA (si-Cntrl), respectively (Fig 4F and 4G). The transcriptionally active nature of the chromatin was determined by quantifying the levels of viral mRNA in PRMT5 depleted cells compared to the control cells. Interestingly, BCBL-1 cells with depleted PRMT5 showed significantly higher levels of almost all the viral transcripts compared to the control cells (Fig 4D). Furthermore, PRMT5 depleted iSLK.219 cells also showed an overall increase, although lesser fold than BCBL-1 cells, in the number of viral transcripts (Fig 4E). This suggested a restrictive role of PRMT5 in regulating KSHV gene expression.

Next, we wanted to determine whether the levels of symmetrically methylated H4R3 chromatin were reduced in those PRMT5 depleted cells. To achieve this, we performed H4R3me2s ChIP on both; BCBL-1 and iSLK.219, control and PRMT5 depleted cells and quantified the symmetric methylation of H4R3 at representative viral gene promoters and genomic regions (Fig 4B). The levels of H4R3me2s in PRMT5 depleted cells were calculated relative to the control cells, which are represented by light grey bars in while the darker grey bars indicate PRMT5 knock-down cells (Fig 4H and 4I). In both the cell lines, PRMT5-depletion correlated with a significant decrease in the amount of H4R3me2s at those representative regions (Fig 4H and 4I). These results suggest that the symmetric dimethylation of H4R3 on the viral genome is dependent upon the expression of PRMT5 and depleting PRMT5 results in a loss of the H4R3me2s modification. Thus, PRMT5 knockdown of KSHV-infected cells impairs H4R3me2s while simultaneously upregulating viral gene transcription.

### ORF59 binding to the viral chromatin displaces PRMT5 from the chromatin

**Fig 5:**
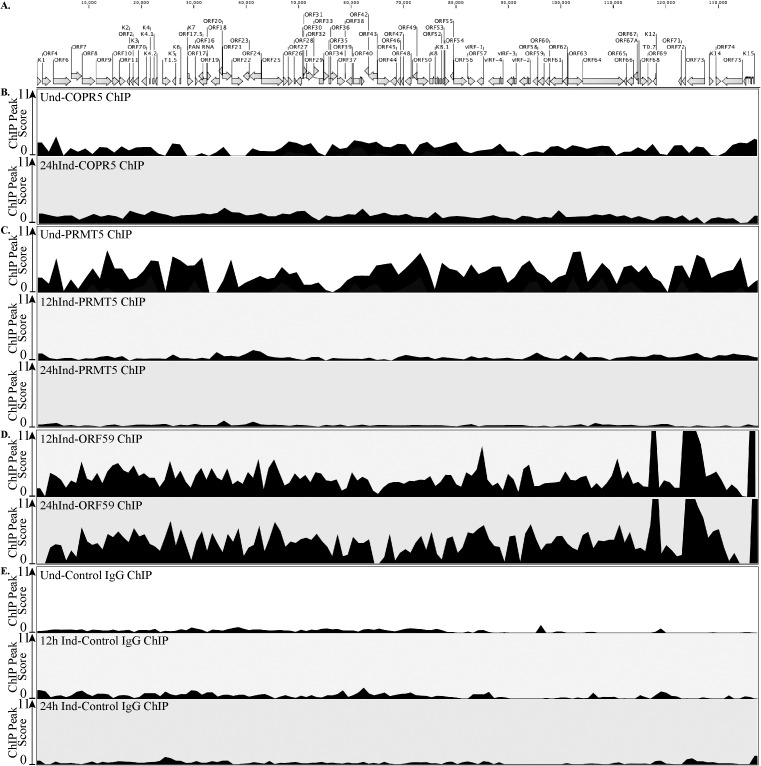
ORF59 binding to the viral genome during reactivation reduced the levels of PRMT5 bound to the viral chromatin. **A.** Schematic of the KSHV genome with ORFs. **B-E.** Uninduced, 12h Ind or 24h Ind (as indicated) TRExBCBL1-RTA cells were harvested, fixed, chromatin was sheared and then precipitated using specific anti-COPR5, anti-PRMT5, anti-ORF59, or anti-IgG antibodies. ChIP DNA was isolated and used to prepare sequencing libraries which were analyzed on the Illumina NextSeq500 instrument. Reads were mapped to the KSHV genome and significant ChIP peaks were identified using “ChIP Seq” tool of CLC Genomics Workbench and the ChIP peaks are presented as peak score. **B.** COPR5 binding on viral chromatin during uninduced (Und) and 24h induced (24h Ind) cells. **C.** PRMT5 binding on viral chromatin during uninduced (Und), 12 and 24h induced (12h Ind, 24h Ind) cells. **D.** ORF59 binding on viral chromatin during 12 and 24h induced (12h Ind, 24h Ind) cells. **E.** Control-IgG ChIP was performed on latent, 12h induced, and 24h induced cells and the reads were mapped to the KSHV genome and analyzed by ChIP-peak calling software to show protein binding specificity.

The reduction of H4R3me2s marks on the viral chromatin during lytic reactivation suggests a dynamic state of arginine methylation that can be altered to favor viral transcription/replication during lytic reactivation. The reduction of H4R3me2s marks at various viral promoters shown in the PRMT5 knockdown cells implies that the presence of PRMT5 on the chromatin can be correlated with the levels of H4R3me2s marks. Taking into consideration PRMT5’s association with ORF59 and its depletion leading to transcriptional activation, we compared the levels of PRMT5’s association to the viral chromatin before, and following lytic induction. To test this, TRExBCBL1-RTA cells were harvested at uninduced, 12h induced or 24h induced time points and used for PRMT5 and ORF59 ChIP-Seq analysis. (Due to the insufficient quantity of ORF59 ChIP DNA isolated from uninduced samples, ORF59 ChIP-Seq was only performed on the 12h and 24h induced cells). PRMT5 showed enriched binding across the latent viral genome, however; upon reactivation at both, 12h and 24h the peaks were diminished suggesting a lower binding of PRMT5 after induction (Fig 5C). Not surprisingly, ORF59 was enriched at numerous loci on the viral genome at both, 12h and 24h post-induction (Fig 5D). Furthermore, in previous studies we tested ORF59’s binding to chromatin in the presence of replication inhibitor, PFA. When replication was inhibited, although perhaps slightly less than control-treated cells, ORF59 still showed significant enrichment at several viral loci (Fig. C in S1 Text).

This demonstrated an important link between ORF59 binding and chromatin structure modulation. The accumulation of ORF59 yet reduction in PRMT5 binding to the viral chromatin suggested a mechanism where ORF59 is responsible for displacing PRMT5 from the chromatin. Interestingly, PRMT5 binds to the chromatin through a ligand, cooperator of PRMT5 (COPR5) [40]. COPR5 functions as a linker molecule that causes PRMT5 to preferentially modify H4R3 residues [40]. To test if PRMT5 is displaced from the chromatin by the detachment of this linker molecule, we also performed COPR5 ChIP Seq on uninduced, and 24h induced TRExBCBL1-RTA cells. In contrast to the PRMT5 ChIP Seq results, COPR5 binding to the viral chromatin remained similar after induction (Fig 5B), confirming that displacement of PRMT5 from COPR5 binding alters symmetric methylation of H4R3. Despite the fact that ORF59, PRMT5, and COPR5 are all DNA-binding proteins, ChIP-seq performed with control-IgG on uninduced, 12h induced, and 24h induced cells did not show any particular peak on the viral genome confirming the specificity of these ligands in chromatin immunoprecipitation (Fig 5E).

### ORF59 competitively disrupted PRMT5’s binding from its ligand

**Fig 6:**
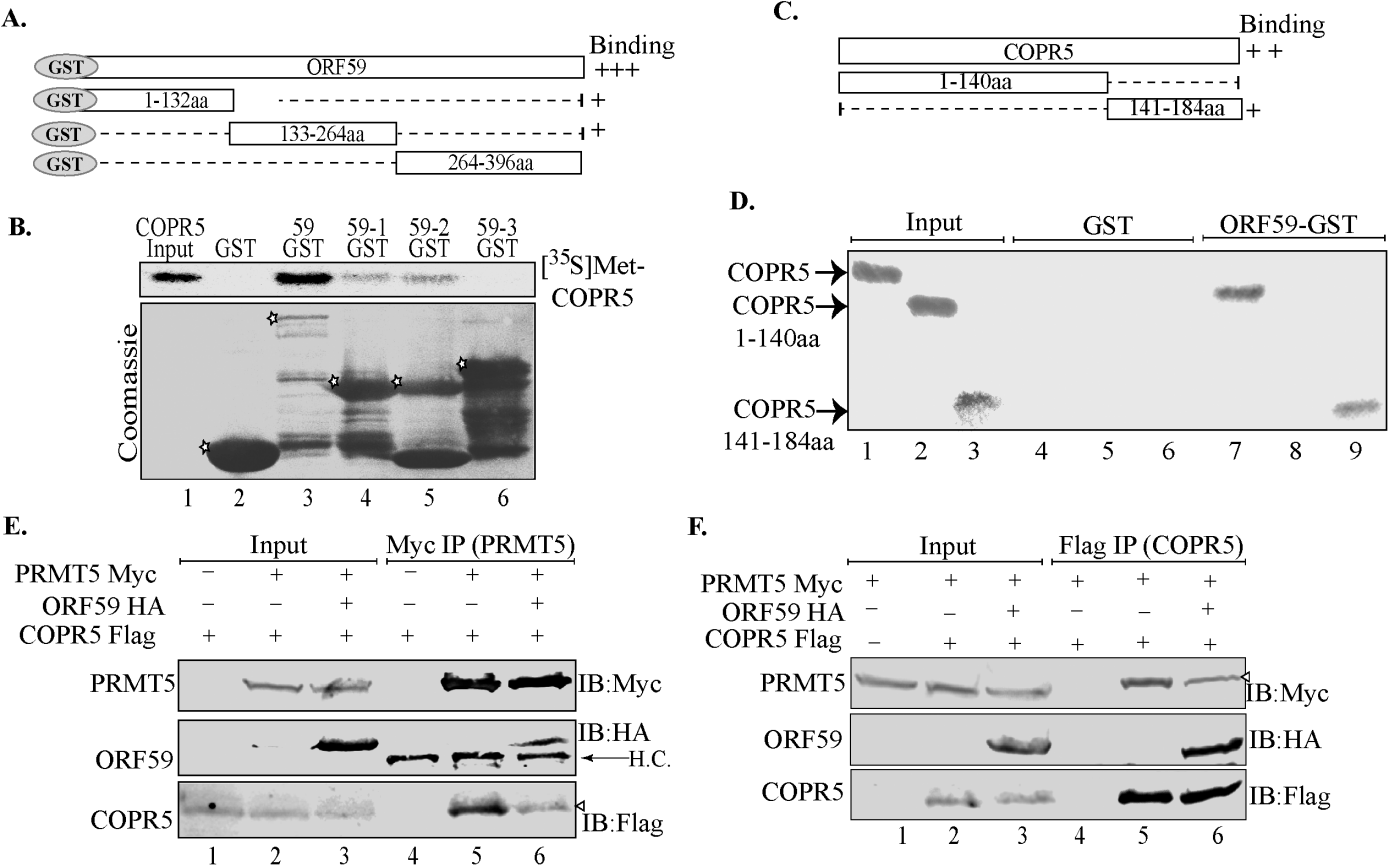
ORF59 competitively disrupts PRMT5’s binding with its binding ligand, COPR5. **A.** Schematic representation of GST-fused ORF59 and its truncation mutants. **B.** *In vitro* translated ^35^S-methionine labeled COPR5 was subjected to a binding assay with ORF59-GST and its truncation mutants, 59–1 (1-132aa), 59–2 (133aa-264aa) and 59–3 (264-396aa). Full-length ORF59 bound most strongly to COPR5 although ORF59-1 and ORF59-2 also associated with COPR5 with lower affinity (compare lane 3 with lane 4 and 5). Asterisks in the coomassie panel indicate control GST (lane 2) GST-fused ORF59 (lane 3) and its truncations (lanes 4–6). **C.** Schematic representation of COPR5 with its histone binding truncation (1-140aa), and PRMT5 binding truncation (141-184aa). **D.** *In vitro* translated ^35^S-methionine labeled COPR5 and its truncations were subjected to binding with control GST (lanes 4–6) or GST fused ORF59 (lanes 7–9). Lanes 1–3 show input levels of *in vitro* translated COPR5 segments. Full length COPR5 and the PRMT5 binding domain of COPR5 (141-184aa) bound to the GST fused ORF59 but not the control GST (compare lanes 7 and 9 to 4 and 6). **E.** PRMT5-Myc, ORF59 HA, and COPR5-Flag were cotransfected and the lysates were immunoprecipitated with anti-Myc antibody to precipitate PRMT5, which precipitated COPR5, as expected (lane 5). However, in the cells expressing ORF59 the association between PMRT5 and COPR5 was reduced, lane 6. ORF59 immunoprecipiated from the complex confirming its association with PRMT5 (lane 6, IB:HA panel). **F.** Competitive binding was also performed by immoprecipitating COPR5 in presence of of ORF59 (lanes 3). The level of COPR5 bound PRMT5 was reduced (compare lanes 5 and 6).

To study the mechanism by which ORF59 could deplete PRMT5 binding from the viral genome in more detail, we tested the binding between ORF59 and the PRMT5-chromatin linker molecule, COPR5. To this end, we created constructs to mimic previously described regions of COPR5: 1-140aa, and 141-184aa. The first larger segment (1-140aa) corresponds to the linker-function of COPR5 and associates with histones whereas the smaller C-terminal truncation of COPR5 (141-184aa) is important for binding to PRMT5. Displacement of PRMT5 from the chromatin prompted us to test whether ORF59 was disrupting the association of PRMT5 with its linker, COPR5. Thus, we performed an *in vitro* binding assay using GST-fused ORF59 protein with ^35^S-methionine labeled, *in vitro*-translated COPR5. First, we determined the binding region of ORF59 by using ORF59 full length and its truncations used previously (Fig 6A). Equal amounts of *in vitro*-translated COPR5 were added to the binding reaction with each of the GST constructs and a representative amount of input is shown (Fig 6B, lane 1). While control-GST showed no association with COPR5 (Fig 6B, lane 2), ORF59 full length showed strong binding (Fig 6B, lane 3). Notably, ORF59-1, ORF59-2 showed some binding but segment 3 (ORF59-3) did not show any binding (Fig 6B, compare lanes 4, 5 with 6). Next, we determined the ORF59 binding domain in COPR5 by using *in vitro* translated COPR5 and its truncations (Fig 6C) with full-length ORF59-GST. Binding was compared with their respective inputs (Fig 6D, lanes 1–3). ORF59-GST interacted with full-length COPR5 and remarkably only the C-terminal region of COPR5 141-184aa (Fig 6D, lanes 7 and 9). The two proteins associated specifically as the control GST did not show bindings (Fig 6D lanes 4–6). The binding of ORF59 to the same domain of COPR5 required for recruiting PRMT5 (141-184aa), but not to the histone-linking region (1-140aa), suggested a mechanism in which ORF59 disrupts the binding of PRMT5 bound to COPR5. This was tested with competitive Co-IPs by expressing ORF59 in cells with COPR5 and PRMT5. In other words, the ability of PRMT5 to bind COPR5 (and vice-versa) was analyzed in the presence or absence of ORF59. In the first set, immunoprecipitation of PRMT5-Myc confirmed its association with COPR5 (Fig 6E, lane 5). However, the presence of ORF59-HA reduced the amounts of co-precipitating COPR5 with PRMT5 (Fig 6E, IB:Flag panel, compare lane 6 with lane 5). The levels of COPR5 in the lysates were comparable (Fig 6E, IB:Flag panel-input lanes, compare lanes 1–3).

We also attempted a reverse co-IP with COPR5 for assaying its binding to PRMT5 in the presence of ORF59 (Fig 6F). Detection of PRMT5 immunoprecipitating with COPR5 confirmed their binding (Fig 6F, lane 5, IB:Myc panel). Presence of ORF59 decreased COPR5’s binding with PRMT5 (Fig 6F, IB:myc, compare lanes 5 and 6). These co-precipitations demonstrate that expression of ORF59 competitively disrupts PRMT5’s association with COPR5 to assist in facilitating structural chromatin changes that favor lytic replication.

### Expression of ORF59 was sufficient to reduce symmetric methylation on the viral genome

ORF59 is an essential protein for lytic viral replication; however, most of the studies were done in presence of a robust viral transcactivator protein, RTA, which is necessary and sufficient to facilitate lytic reactivation [54, 55]. Therefore, we wanted to investigate the role of ORF59 in modulating chromatin structure in cells lacking this overarching factor, iSLKTet-RTA-Bac16-RTASTOP [56].

**Fig 7:**
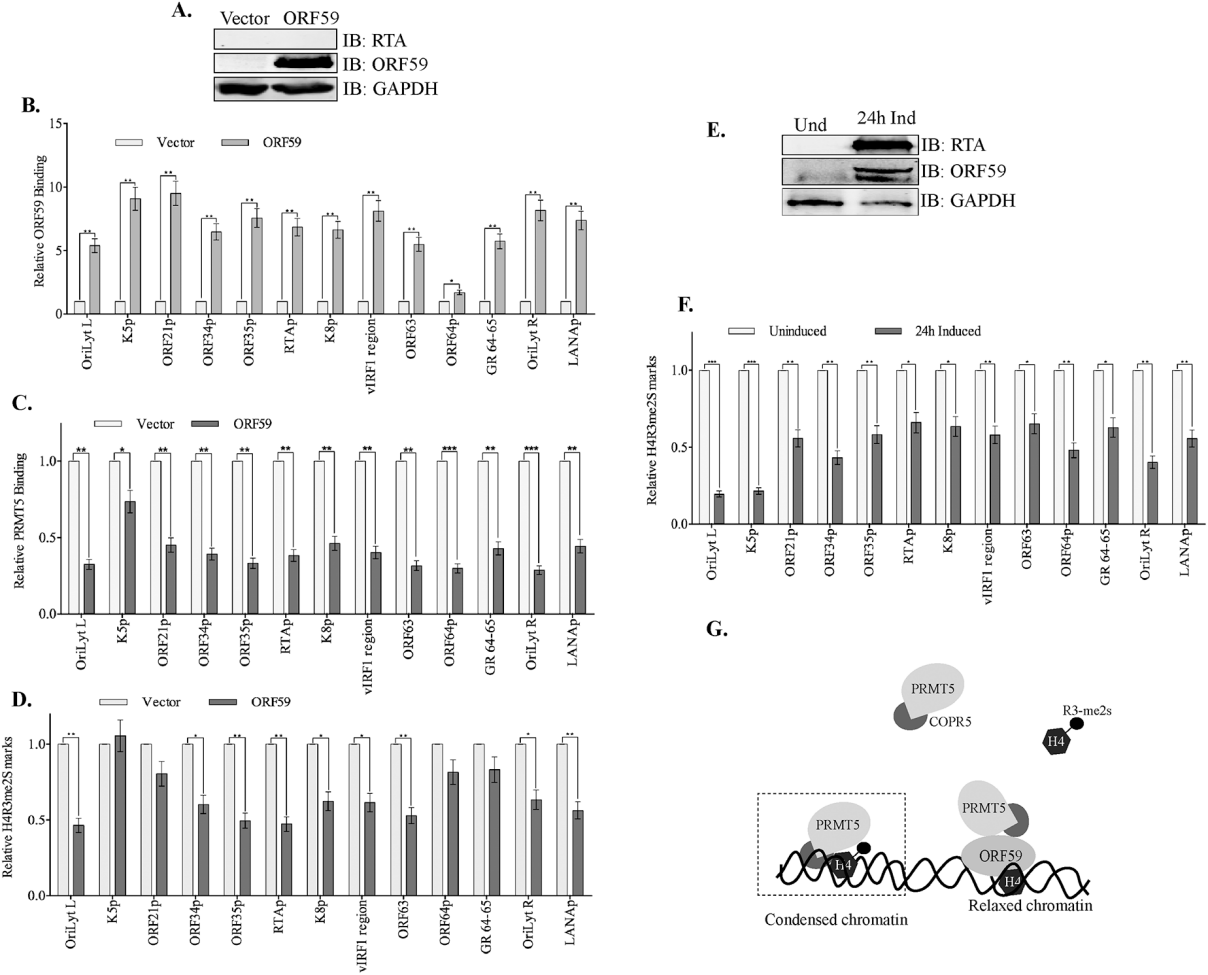
ORF59 orchestrates chromatin modification independent of RTA. **A.** iSLK-BAC16-RTASTOP cells were transfected with ORF59 or control vector and the expression was confirmed by WB. **B.** Chromatin from the iSLK-BAC16-RTASTOP with ORF59 or vector transfected cells were immunoprecipitated with anti-ORF59 antibody and analyzed for its binding at various viral promoters. ORF59 overexpression in these RTA-depleted cells resulted in specific binding of ORF59 to the viral promoter targets when compared to the cells without ORF59 expression. **C.** ChIP with PRMT5 was performed to determine the binding of PRMT5 in presence of ORF59 at those selected regions of the viral genome, which showed reduced binding, as detected in the lytically reactivated cells. **D.** ChIP assay with anti-H4R3me2s antibody was performed to determine the levels of chromatin bound to symmetrically methylated H4R3. In alliance with PRMT5 being depleted, the levels of H4R3me2s are reduced at the viral promoters in the presence of overexpressed ORF59. **E.** iSLK-BAC16-RTASTOP cells were either un-induced, or lytically reactivated by doxycycline for 24h, expressed RTA and ORF59. **F.** These doxycycline induced or uninduced iSLK-BAC16-RTASTOP cells were subjected for ChIP with anti-H4R3me2s antibody for detecting chromatin bound to H4R3me2s chromatin. Consistent with our previous results, H4R3me2s bound chromatin was less prevalent at viral promoters during induction as compared to the uninduced, latent cells. **G.** Proposed model of the mechanism by which ORF59 depletes PRMT5 binding from the chromatin to facilitate the relaxation of viral chromatin and gene transcription.

iSLKTet-RTA-Bac16-RTASTOP cells were transiently transfected with ORF59 for the detection of chromatin modifications using ChIP assays. Expression of ORF59 and absence of RTA in iSLKTet-RTA-Bac16-RTASTOP cells was confirmed by immune detection (Fig 7A). We then determined the enrichment of ORF59 on the viral chromatin across a number of important loci including *OriLyt* region and the RTA promoter region (Fig 7B). The amounts of ORF59 bound to the viral promoters were calculated relative to the control-plasmid transfected iSLKTet-RTA-Bac16-RTASTOP cells. The data showed varying levels of ORF59 binding despite the absence of RTA on the representative regions of the viral genome (Fig 7A). Next, we used the same cells to determine the association of PRMT5 to the viral chromatin. This revealed a consistent pattern of lower PRMT5 bound at those representative sites (Fig 7C, darker bars represent PRMT5 binding in presence ORF59). Strikingly, the expression of ORF59 in those iSLKTet-RTA-Bac16-RTASTOP cells was enough to trigger significant reductions of H4R3me2s marks on the viral chromatin at various viral promoter regions and the *OriLyt* (Fig 7D). In fact, the reduction in symmetrically methylated H4R3 due to ORF59 expression was fairly comparable to those obtained from iSLKTet-RTA-Bac16RTASTOP cells induced for lytic replication with doxycycline for RTA expression (Fig 7F). These cells showed a robust expression of RTA and ORF59 (Fig 7E). These results suggest a model in which PRMT5 binds to the latent viral chromatin to symmetrically methylate the H4R3 but the expression of ORF59 represses H4R3me2s levels by altering PRMT5’s association with COPR5 in order to alter the chromatin landscape (Fig 7G).

### Reduction in H4R3me2s corresponds with an increased level of activating marks

**Fig 8:**
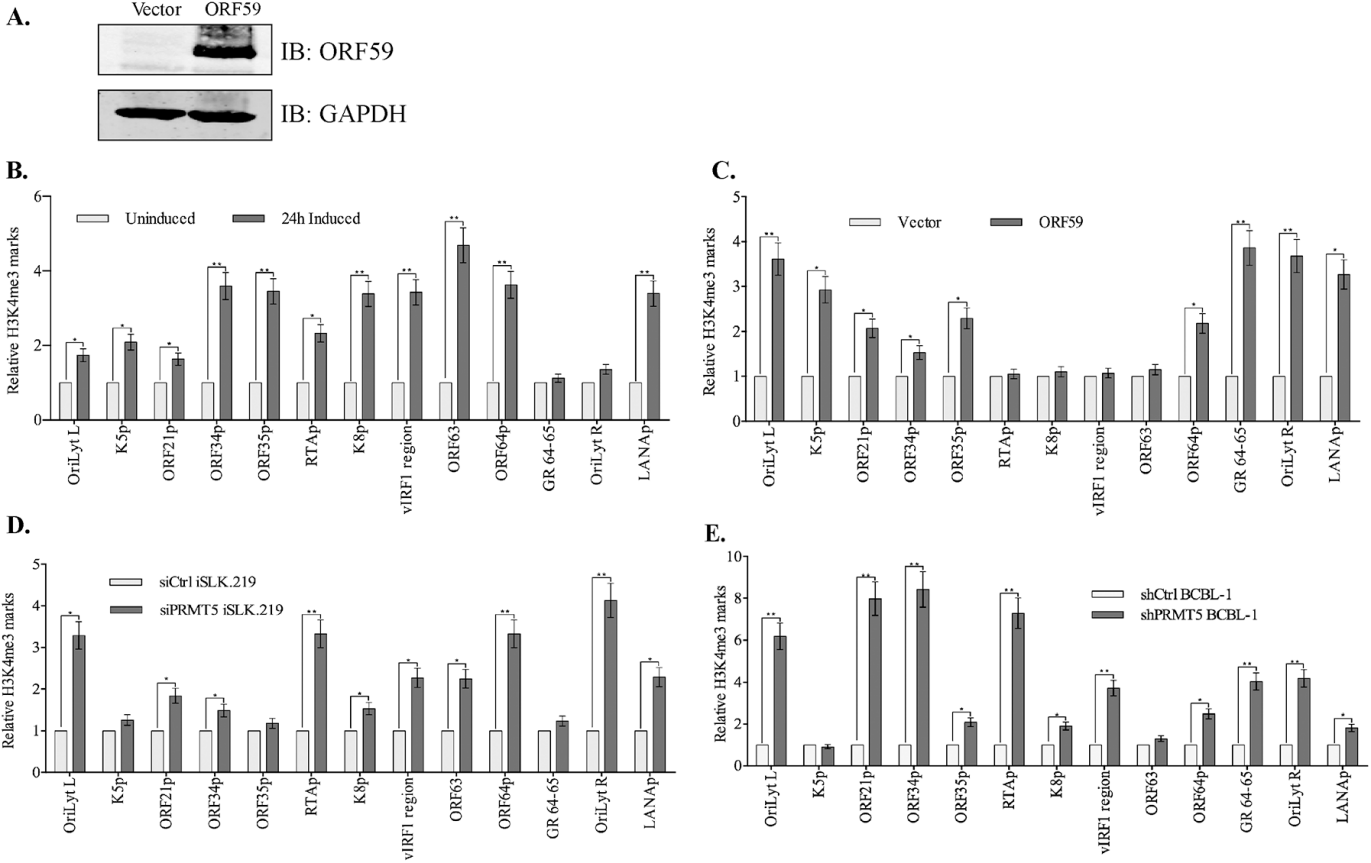
Loss of H4R3me2s corresponds with an enrichment of H3K4me3 on viral chromatin. **A.** iSLK-BAC16-RTASTOP cells were transfected with ORF59 or control vector plasmids to express only ORF59 in these latent cells. **B.** iSLK-BAC16-RTASTOP were induced with doxycycline for 24h, fixed, and chromatins were sheared and immunoprecipitated with anti-H3K4me3 antibody. Congruent with previous reports, the levels H3K4me3 bound chromatin were enriched early during lytic reactivation at representative regions. **C.** iSLK-BAC16-RTASTOP cells expressing ORF59 were subjected for ChIP with anti-H3K4me3 antibody, which showed an enrichment of H3K4me3 at various viral promoters calculated relative to the vector transfected cells. **D.** iSLK.219 cells transiently transfected with control or PRMT5 siRNA to deplete PRMT5 were subjected for ChIP with anti-H3K4me3 antibody. Relative amounts of H3K4me3 bound chromatin was enhanced ion In PRMT5 depleted cells as compared to the control cells. **E.** Similarly, BCBL-1 cells depleted for PRMT5 by stable transducing specific shRNA lentiviral vector showed an enhanced level of H3K4me3 bound chromatin as compared to control sh RNA lentiviral transduced cells shown at representative regions.

The change in H4R3me2s marks and expression of viral transcripts due to PRMT5 depletion and ORF59 expression prompted us to evaluate whether reduction in the levels of H4R3me2s leads to an alteration of other epigenetic marks on the chromatin favoring lytic replication. To this end, we evaluated the levels of H3K4me3, a well-known activating mark on the viral chromatin, previously shown to be enriched during lytic reactivation [20, 22, 57]. Interestingly, studies have shown that the presence of H4R3me2s mark inhibits efficient tri-methylation of H3K4 residues to preserve a heterochromatic landscape [34, 58]. Our results confirmed a decrease of these prohibitive H4R3me2s marks at various viral promoters in the presence of ORF59, therefore it was necessary to test whether this led to an increase in the abundance of activating, H3K4me3 marks on those sites. To this end, chromatin immunoprecipitation with anti-H3K4me3 antibody was performed on cells with a series of different cellular/viral conditions. iSLKTet-RTA-Bac16-RTASTOP cells were induced for RTA expression by the addition of doxycycline for the precipitation of viral chromatin bound to H3K4me3. Relative levels of H3K4me3 bound chromatin after lytic induction (Fig 8B, dark bars) were calculated by normalizing with the levels in uninduced cells (Fig 8B, dark bars) (a relative fold change of 1 represents the H3K4me3 levels on viral genome before lytic induction (Fig 8B)). Consistent with previous findings [20, 22, 57, 59], lytic induction increased the abundance of activating, H3K4me3 marks on the viral chromatin, shown on the represented targets (Fig 8B). In addition, we used the same cell line, iSLKTet-RTA-Bac16-RTASTOP for detecting the role of ORF59 on H3K4 methylation levels in absence of RTA. The expression of ORF59 in these iSLKTet-RTA-Bac16-RTASTOP cells was confirmed by immune detection (Fig 8A). Chromatin bound to H3K4me3 histone showed a significant enrichment at various viral promoters in cells expressing ORF59 as compared to the vector-transfected cells (Fig 8C), which corroborated with reduction in the levels of H4R3me2s marks. These results confirmed that ORF59 by itself was capable of inducing changes in chromatin structure resembling those that occur during lytic reactivation. Next, we wanted to determine the levels of H3K4me3 bound chromatin in cells depleted with PRMT5, which showed a significant decrease in levels of H4R3me2s bound chromatin. Not surprisingly, the levels of H3K4me3 bound chromatin were increased in PRMT5 depleted iSLK.219 cells as compared to the control cells (Fig 8D). Similarly, BCBL-1 cells depleted for PRMT5 by shRNA showed significant increase in H3K4me3 bound chromatin compared to the control cells (Fig 8E). This was consistent with the previous observation that H4R3me2s has an inhibitory effect on H3K4me3 and removal H4R3me2s leads to an enrichment of active chromatin mark, H3K4me3 [34, 58, 60].

### ORF59 is necessary for efficient gene expression during reactivation

**Fig 9:**
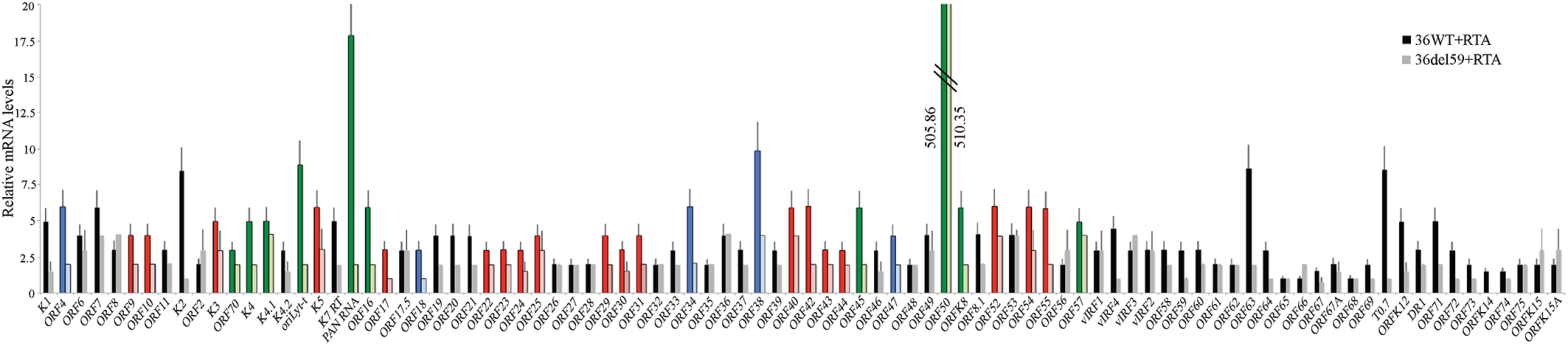
Expression of ORF59 triggers expression of viral genes. 293LBac36WT or ORF59 deleted (293LBac36Δ59) cells were transfected with RTA to induce lytic reactivation. Cells were harvested 48h post transfection cells for the detection of viral mRNA in a real-time qPCR assay. Expression of many viral genes were reduced in ORF59 deleted cells and the genes highlighted in green are immediate early, blue-early genes and red are the late genes.

ORF59 has been previously characterized to be essential for efficient viral DNA replication, but given the role we describe here in regards to chromatin structure modulation, we wanted to investigate the effects of ORF59 protein on the transcription of viral genes. To this end, we utilized previously described KSHV Bacmid deleted with ORF59 (Bac36Δ59) in 293L cells [50]. We harvested RNA from wild type (293L-Bac36WT) and ORF59 deleted (293LBac36Δ59) cells transiently transfected with RTA for the induction of lytic cascade. The levels of RTA were comparable between these two sets, RTA transcripts were 505 and 510 folds in wt and ORF59 deleted cells, respectively (Fig 9). The levels of the viral genes transcripts in 293L-Bac36WT with RTA cells were calculated by comparing with the control vector transfected, 293L-Bac36WT cells. Similarly, the viral genes transcripts in 293LBac36Δ59 expressing RTA were calculated by taking vector transfected, 293LBac36Δ59 cells, as control. The relative fold change of viral genes with RTA from 293L-Bac36WT and 293LBac36Δ59 were plotted and showed that in cells lacking ORF59, the mRNA copies of many viral genes were reduced (Fig 9). We analyzed the effects of ORF59 on the expression of immediate early, early, and late genes. Immediate early (IE) genes, displayed in green, showed consistently higher levels of transcripts in the Bac36WT as compared to the cells deleted for ORF59 (Fig 9, Dark green-Bac36WT and light green-ORF59 deleted). A handful of early genes, shown in blue shade, also showed dependence on ORF59 for their expression and these include ORF59: ORF4, ORF18, ORF34, ORF38, and ORF47 (Fig 9, Dark blue- Bac36WT and light blue- Bac36Δ59). Interestingly, a greater number of late gene transcripts, represented by the red bars (Fig 9, Dark red-Bac36WT and light red-ORF59 deleted) were affected by the depletion of ORF59, suggesting that ORF59 plays an important role in controlling viral chromatin landscape for gene transcription.

## Discussion

ORF59 encoded processivity factor is one of the viral lytic proteins that helps the viral DNA polymerase with processivity activity and is essential for the productive replication of the viral genome [42, 44, 46, 48, 50]. We previously showed that phosphorylation of ORF59 with viral kinase is essential for the processivity function and virion production [50]. ORF59 was classically viewed as DNA replication protein but it has been shown to regulate additional processes involved in promoting lytic reactivation. These include, binding to cellular helicases, Ku70/Ku86 to impair non-homologous end joining of replicated DNA [51], and binding and degrading PARP-1 to alleviate the repressive effects of PARP-1 on viral lytic replication [52]. These additional functions prompted us to identify the proteins interacting with ORF59 during lytic replication to have a clear understanding of its role in the viral life cycle. Using a Flag epitope tagged version of ORF59 in BAC16 was advantageous for identifying the proteins specifically associating with ORF59, including a large number of viral and cellular proteins. Among them we observed a chromatin modifying protein, PRMT5, as a significant ORF59 binding protein because ORF59 is detected early during reactivation and is critical for processes involved in DNA replication [24, 49, 50, 61, 62].

PRMT5, an arginine methyltransferase, was characterized as a transcriptional repressor bound to the chromatin of a promoter region in conjunction with multiple repressive marks including hypoacetylated H3 and H4 and lysine methylated H3K9 residue [41]. Additional reports confirmed PRMT5 to be a chromatin binding protein specifically on a transcriptionally repressive region in cooperation with other repressive complexes or transcription factors including Blimp1, Snail, BRG1 and hBRM [63–66]. Our immunoprecipitation and localization studies revealed that ORF59 and PRMT5 interact in the nucleus of KSHV infected cells during lytic reactivation, which was consistent throughout multiple cell lines. After confirming the association between ORF59 and PRMT5, we postulated that ORF59, being a protein required for lytic DNA replication, which in turn occurs on open chromatin, might alter the repressive functions of PRMT5. To better understand the nature of the interaction of these two proteins, we determined the domains required for their association in *in vitro* binding assays. The N-terminal domain of ORF59, 1-132aa interacted most strongly with PRMT5. Interestingly, the N-terminal domain of ORF59 is the domain through which ORF59 homodimerizes and interacts with the viral DNA polymerase [44, 46, 47, 49]. On the other hand, the interaction domain of PRMT5 that binds to ORF59 mapped to its middle domain from residues 210-420aa. The middle segment of PRMT5 possesses the methyltransferase activity, which prompted us to generate a clone containing the catalytic domain of the middle segment for assaying its binding with ORF59. Intriguingly, ORF59 was able to interact with this small domain of PRMT5, indicating that ORF59 could be involved in modulating the methyltransferase activity of PRMT5.

A previous report showed that arginine methyltransferases could associate with viral proteins to methylate them and alter their function [67]. KSHV latent protein, LANA associates with an arginine methyltransferase type I, PRMT1, which methylates arginine residues in an *asymmetric* fashion [67–69]. This demonstrated that KSHV proteins are not only associated with cellular arginine methyltransferases, but are indeed post-translationally modified by them to alter specific functionality. This begged the obvious question of whether ORF59 is arginine-methylated by associating with PRMT5. To investigate this, we used a methylation prediction software, MASA (Methylation site based on the Accessible Surface Area) available at http://masa.mbc.nctu.edu.tw/predict.php [70], which found that only a single arginine, R384 could potentially be methylated (Fig. D in S1 Text) suggesting that its association with PRMT5 may be required for its function. However, we did not pursue the methylation of ORF59 in this report [71].

As mentioned earlier, PRMT5 symmetrically dimethylates the H4R3 residues of histone H4 to form a transcriptionally repressive chromatin, so we determined the levels of H4R3me2s marks on latent viral genome by performing a sequencing of the H4R3me2s bound DNA. This showed a significant enrichment throughout the latent genome while the levels of symmetrically methylated H4R3 on the viral chromatin were reduced upon reactivation. This coupled with the similar levels of H4 occupancy on the viral genome confirmed that histone arginine methylation is differentially regulated during different phases of the viral life cycle.

The dynamic nature of the chromatin landscape of the viral genome facilitates rapid change for controlling the KSHV lifecycle phases. The viral genome persists in a highly ordered chromatinized episome within the host cells and the associated chromatin is subject to modifications for the concealment or exposure of particular genes for transcription as needed during latency or the lytic reactivation cascade [20, 21, 25, 26]. The availability of genes for transcription is regulated by the presence or absence of a variety of multiple histone-tail modifications, including H4R3me2s [28]. To date, most of the reports on H4R3me2s methylation show its association with transcriptionally repressed gene [60, 72]. However, one study found PRMT5 to be over-expressed in transformed chronic lymphocytic leukemia (B-CLL) cells with elevated levels of global H4R3me2s marks [73]. Interestingly, this enrichment in H4R3me2s also resulted in the transcriptional repression and subsequent downregulation of a tumor suppressor family of genes, pRB [73]. Another study conducted using EBV positive cells as a model tested the effects of PRMT5 mediated gene repression [74]. They used a PRMT5 inhibitor to determine the importance of repressive epigenetic marks in context of EBV tumors and concluded that PRMT5 was critical for B cell transformation and malignancies because the overexpression of PRMT5 helped to silence the tumor suppressor genes [74]. Another report generated a sophisticated *in silico* modeling procedure to analyze the ChIP-Seq data from 20 different histone methylations concluded that in the context of simultaneous activating and repressive marks, H4R3me2s is one of the most abundant repressive marks associated with gene silencing [60]. Thus, after confirming the association between ORF59 and PRMT5, the reduction of H4R3me2s marks (that PRMT5 is responsible for bestowing) on the viral genome during lytic reactivation appeared in agreement with its role and we elucidated a mechanism by which ORF59 might be directly or indirectly facilitating these changes to the viral chromatin.

It was interesting to note that the binding of ORF59 to the viral genomic regions reduced the levels of chromatin bound PRMT5, which was primarily due to a change in specificity of PRMT5 with its linker COPR5 [40]. PRMT5’s binding specificity to the chromatin is determined in large by the co-factors it associates with and we found that ORF59 disrupted the association between PRMT5 and the linker molecule COPR5 that tethers PRMT5 preferentially to the chromatin for symmetrically methylating the H4R3 residue [40]. In essence, presence of ORF59, which expresses during lytic reactivation, disrupts the binding of PRMT5 to the chromatin by competitively causing its detachment from its linker molecule, COPR5. Notably, the binding of COPR5 to the viral chromatin did not appear to change significantly which in conjunction with the fact that ORF59 did not associate with the histone H4 binding region of COPR5 1-140aa, suggests that ORF59 does not disrupt that function but rather blocks PRMT5’s specific affinity for histone H4. Moreover, ORF59 appears to be directly involved in regulating these lytic reactivation-permissive changes to the chromatin as the KSHV genome lacking immediate early genes (RTA-stop cells) showed similar reduction in chromatin bound PRMT5 and H4R3me2s marks at the viral promoters when supplemented with ORF59.

The reduction of the levels of PRMT5 bound to the chromatin led to a reduction in the symmetrically methylated H4R3 and gene activation. Similarly, it was previously seen in cancer cells in which treatment with a drug, AS1411 (a quadruplex-forming oligonucleotide aptamer) led to the decreased association of PRMT5 with known gene promoters (cyclin E2, tumor suppressor genes)[29, 75]. As a result of this depletion, PRMT5 regulated genes regained their activity [75]. Thus, the presence or absence of PRMT5 on the chromatin can be linked to the levels of repressive marks and transcriptional activity, even on the KSHV genome.

Cross-talk between different histone modifications to epigenetically regulate gene expression has been extensively studied in recent years and although still imperfectly understood, it is known that the relative presence or absence of modifications at different residues of the histone tails influences precise transcription patterns [76]. Indeed, our experiments suggest that there is a cross talk between repressive H4R3me2s marks and activating H3K4me3 marks. When H4R3me2s was reduced at various viral promoters (due to PRMT5 depletion or ORF59 overexpression), an enrichment of H3K4me3 at the same promoters was detected (Fig 8). This corroborated with a previous report, which showed that H4R3me2s sterically inhibits H3K4 methyltransferase activity to promote gene silencing [34]. This study on an analogous Type II arginine methyltransferase (PRMT7) implicated the presence or absence of methyltransferase in the prevalence and levels of the corresponding histone marks [34]. Yao et al. determined that overexpression of type II arginine methyltransferase, PRMT7 in cancer cells inhibited the expression of a specific gene promoter by causing an elevation of H4R3me2s marks, shown in conjunction with reduced H3K4me3, H4ac, and H3ac [58]. Furthermore, knockdown of PRMT7 led to a restoration of gene expression by repressing the levels of H4R3me2s and increasing the H3K4me3 and acetylation of H4 [58]. This strongly confirms that regulating the activity of arginine methyltransferases can cause epigenetic changes to influence gene expression.

Other KSHV proteins are capable of modulating viral chromatin during infection, which includes LANA and RTA [19]. RTA has been previously shown to not only autoactivate its own promoter, but also transactivate other viral gene promoters including (but not limited to) vIL-6, PAN (polyadenylated RNA), ORF57, and ORF59 [77–80]. Due to the known robust effects of RTA, which is also present during viral reactivation, we wanted to determine the impact of ORF59 on KSHV chromatin structure and viral gene expression. Our data showed that ORF59 overexpression, independent of RTA, led to a reduction of PRMT5 binding, loss of H4R3me2s marks, and enrichment of H3K4me3 marks at various viral promoters signifying the role ORF59 in these alterations. The downstream effects of changes to the chromatin structure were evident by the modulated transcription of the lytic viral genes, meaning those structural chromatin changes orchestrated by ORF59 ultimately affect lytic gene transcription. Importantly, the transcription of the lytic viral genes in the absence of ORF59 was not as efficient in ORF59-depleted cells as compared to the wild-type cells. Therefore, this study clearly shows that the lytic protein ORF59 has multiple functions during viral reactivation to facilitate efficient gene transcription for viral replication.

## Materials and Methods

### Cell culture

293T and 293L (ATCC, Manassas, VA) cells were grown in high-glucose Dulbecco's modified Eagle's medium (DMEM) supplemented with 8% bovine growth serum (HyClone, Logan, UT), 2 mM l-glutamine, 25 U/ml penicillin, and 25 μg/ml streptomycin. Additionally, 293Ls harboring any of the following: BAC16WT, BAC16 ORF59-Flag, BAC16RTASTOP (generous gift from Dr. Jae Jung), BAC36WT, BAC36Δ50, or BAC36ΔORF59, were cultured in above DMEM supplemented with 50μg/mL hygromycin B. iSLK.219 (generous gift from Dr. Don Ganem), iSLKTet-RTABAC16WT, and iSLKTet-RTABAC16RTASTOP cells (generous gift from Dr. Jae Jung) were maintained in DMEM supplemented with 10% Tet-Free Fetal Bovine Serum with additional 600μg/mL hygromycin B, 400μg/mL G418, 1μg/mL puromycin. iSLK.219 cells with recombinant KSHV BACs were induced by doxycycline.

TRExBCBL1-RTA (generous gift from Dr. Jae Jung) and BCBL-1 (ATCC, Manassas, VA), and cells were grown in Roswell Park Memorial Institute medium (RPMI) supplemented with 8% fetal bovine serum (HyClone, Logan, UT), 2 mM l-glutamine, 25 U/ml penicillin, and 25 μg/ml streptomycin. BCBL-1 transduced with shRNA PRMT5 lentiviral vectors (GE Dharmacon, Lafayette, CO) were selected and maintained on 1μg/mL puromycin. BACmid-containing 293L cells were transduced with indicated lentiviral vectors and maintained on 1μg/mL puromycin. Constituently expressing ORF59-Flag, and dsRedORF59-Flag in 293L cells were generated using a lentivirus system and ORF59-Flag expressing BACmid. pLVX-ORF59-Flag or pLVXdsRed-ORF59-Flag lentiviral vectors were generated by introducing the gene into respective vectors, and transfecting into 293T cells along with the packaging vectors (CMV-dR8.2, pCMV-VSVG) (Addgene, Cambridge, MA) for producing virions. Respective vector was also transfected into 293T cells for producing vector control lentivirus. Collected viruses were added to the target cells for transduction followed by selection with puromycin (2 mg/ml) to obtain a pure population of cells. BAC16 ORF59-Flag was transfected into 293L cells with Metafectene Pro (Biontex Laboratories GmbH, San Diego, CA) as previously described [50] followed by selection with hygromycin to obtain cells maintaining the KSHV BACs. The selection in both cell lines was monitored with GFP or RFP signals encoded by the lentivirus and the BACmid. All cultures were incubated at 37°C in a humidified environment supplemented with 5% CO_2_.

### Plasmids

The following plasmids were generated by PCR amplification and cloning: pLVXORF59-Flag, pLVXdsRedORF59-Flag, pA3M-PRMT5-Myc, pA3F-PRMT5-Flag, pxiORF59-HA, pA3M-COPR5-Myc, pA3F-COPR5-Flag, pLVX-RTA, pGex-ORF59-GST. The following plasmids were then sub-cloned: pA3F-PRMT51-210aa-Flag, pA3F-PRMT5210-420aa-Flag, pA3F-PRMT5420-637aa-Flag, pA3F-COPR51-140aa-Flag, pA3fCOPR5141-184aa-Flag, pGex-ORF591-132aa-GST, pGex-ORF59133-264aa-GST, pGex-ORF59265-396aa-GST. pGIPz shRNA PRMT5 and Control shRNA lentiviral vectors were obtained from commercial source (Thermo Scientific Inc.). Packaging lentiviral vectors were obtained from Addgene.

PRMT5, COPR5 and their truncations were generated by PCR amplification using specific primers listed in Table C in S1 Text and cloning into respective vectors. The integrity of clones was confirmed by sequencing at Nevada, Genomics Center, Reno.

### Generation of Flag-tagged ORF59 BACmid (Bac16-ORF59Flag)

To generate a recombinant KSHV BAC16 with ORF59-Flag tagged, the epitope tag was inserted at the C-terminus of ORF59 by homologous recombination using a two-step “*seam-less*” *gal*K positive/counter selection scheme. First, the *Gal*k-*Kan*R cassette with homologous flanking sequence was PCR amplified using primers with 50bp-homologus sequences (before and after the target sequence) in the sense and anti-sense primers. *Gal*k-*Kan*R cassette was amplified by including 20-nt of homology to the *Gal*k-*Kan*R region. Primers used for Galk-KanR cassette insertion (bold case is homologous to ORF59 locus, lower case is homologous to the *Gal*k-*Kan*R plasmid):

Forward primer: 5’-GATCGTGGGAAGGTGCCCAAAACCACATTTAACCCCCTGATTGACTACAAAGACGATGACGACAAGTGAcctgttgacaattaatcatc-3’,

Reverse primer: 5’-CTGAAGAGCGACAGAGCGCGCTCACTGTCCAGGCGGCACATGGTGctcagcaaaagttcgattta-3’.

The PCR product containing the target sequence was subjected to *Dpn*I digestion, followed by agarose gel purification to remove any residual template plasmid. PCR product was then electroporated into competent *E*. *coli* strain, SW102 containing BAC16. The *Gal*k-*Kan*R cassette containing mutants were selected on chloramphenicol/kanamycin agar plates and correct insertional mutants were confirmed by restriction digestion with BamHI and Southern blot analysis of fragment containing the *Gal*K cassette. The *Gal*K-*Kan*R cassette was replaced by electroporating a double stranded oligo (5’-ACATTTAACCCCCTGATTGACTACAAAGACGATGACGACAAGTGACACCATGTGCCGCCTGGACAGTGAGCGCGCTCTGTCGCTCTTCAG-3’) with homology to the flanking site and plating the bacteria on 2-deoxy galactose (DOG) containing agar plates for counter selection. Correct colonies were screened and subjected to confirmation by southern blot analysis, PCR amplification of the junctions and sequence analysis.

### Antibodies

The following antibodies were used: mouse anti-Flag (M2, Sigma-Aldrich, St. Louis, MO), rabbit anti-Flag (F7425, Sigma-Aldrich, St. Louis), mouse anti-RTA (mouse hybridoma), mouse anti-LANA (mouse hybridoma), mouse anti-Myc (mouse hybridoma), rabbit anti-HA (6908, Sigma-Aldrich, St. Louis, MO), mouse anti-HA12CA5 (sc-57592, Santa Cruz Biotechnology), mouse anti-GFP (G1546, Sigma-Aldrich, St. Louis), mouse anti-GAPDH (G8140, US Biological, Salem MA), and rabbit anti-Myc (SAB4300605, Sigma-Aldrich, St. Louis, MO), goat anti-PRMT5 (C-20, sc-22132, Santa Cruz Biotechnology), mouse anti-ORF59 (generous gift from Dr. Bala Chandran), rabbit anti-Control IgG (sc-2027, Santa Cruz Biotechnology), mouse anti-Control IgG (sc-2025, Santa Cruz Biotechnology), rabbit anti-H4R3me2s (61187, Active Motif, Carlsbad CA), rabbit anti-Histone H4 (#61299 Active Motif), rabbit-anti-PRMT5 antibody (#61001 Active Motif), rabbit anti-control IgG (ChIP grade—Cell Signaling Technology #2729, rabbit anti-COPR5 antibody (Novus Biologicals #NBP2-30884), and additional rabbit-anti ORF59 antibody custom synthesized for our lab by GenScript.

### Co-immunoprecipitations and Western blotting

For overexpression experiments, 293T cells were plated to 70–80% confluency followed by transfecting them with expression vectors by combining PEI, transfection reagent and 150mM NaCl, mixing thoroughly and incubating it for 15 minutes at room temperature. The mixtures were then added onto 70–80% confluent 293T cells and incubated for 6 h at 37°C with 5% CO_2_ before changing the medium to remove PEI. Transfected cells were harvested after 48 h post-transfection for immunoprecipitation assays. Harvested cells were washed with ice-cold PBS and lysed in 0.5 ml ice-cold RIPA buffer (1% Nonidet P-40 [NP-40], 50 mM Tris [pH 7.5], 1 mM EDTA [pH 8.0], 150 mM NaCl), supplemented with protease inhibitors (1 mM phenylmethylsulfonyl fluoride, 1 μg/ml aprotinin, 1 μg/ml pepstatin, 1μg/mL sodium fluoride, and 1 μg/ml leupeptin). Cell debris were removed by centrifugation at 13,000×g (10 min and 4°C), and lysates were then precleared for 1h with rotation at 4°C with 30 μl of Protein A-Protein G-conjugated Sepharose beads. Approximately, 5% of the lysates were saved for input control and remaining was added with 1.0 μg of indicated antibodies to capture the protein by rotating overnight at 4°C. Immune complexes were captured with 30 μl of Protein A-Protein G-conjugated Sepharose beads with rotation for 2h at 4°C. The beads were pelleted and washed three times with RIPA buffer. Input lysates and the immunoprecipitated complexes were boiled for 5–7 min in Laemmli buffer, resolved on SDS-PAGE and transferred onto nitrocellulose membrane (Bio-Rad Laboratories). The membranes were incubated with appropriate antibodies followed by detection with infrared-dyes tagged secondary antibodies and imaged on an Odyssey imager (LICOR Inc., Lincoln, NE).

### Chromatin immunoprecipitation

Chromatin immunoprecipitation was performed as described previously [16]. Briefly, approximately 20 million cells were cross-linked with 1% formaldehyde for 10 min at room temperature, followed by addition of 125 mM glycine to stop the cross-linking reaction. Cells were washed with cold PBS containing protease inhibitors (1 μg/ml leupeptin, 1 μg/ml aprotinin, 1μg/mL sodium fluoride, 1 μg/ml pepstatin, and 1 mM phenylmethylsulfonyl fluoride). Cells were resuspended in 1 ml cell lysis buffer [5 mM piperazine-*N*, *N*′-bis (2-ethanesulfonic acid) (PIPES)-KOH (pH 8.0)-85 mM KCl-0.5% NP-40] containing protease inhibitors, incubated on ice for 10 min followed by centrifugation at 2,500 rpm for 5 min at 4°C to collect the nuclei. Nuclei were resuspended in nuclear lysis buffer (50 mM Tris [pH 8.0]-10 mM EDTA-1% SDS containing protease inhibitors), followed by incubation on ice for 10 min. Chromatin was sonicated to an average length of 700 bp followed by removing the cell debris by centrifugation at 13,000 rpm for 10 min at 4°C. The supernatant containing sonicated chromatin was diluted with ChIP buffer (0.01% SDS-1.0% Triton X-100-1.2 mM EDTA-16.7 mM Tris [pH 8.1]-167 mM NaCl including protease inhibitor). Samples were precleared with a salmon sperm DNA-protein A-protein G Sepharose slurry for 1h at 4°C with constant rotation. The supernatant was collected after a brief centrifugation (2,000 rpm at 4°C). Ten percent of the supernatant was saved for input and the remaining 90% was used for capturing chromatin by rotating the complexes overnight at 4°C using indicated antibodies. The antibody bound chromatin was precipitated by protein A/G slurry. Beads were then washed sequentially three times with a low-salt buffer (0.1% SDS-1.0% Triton X-100-2 mM EDTA-20 mM Tris [pH 8.1]-150 mM NaCl), and twice in Tris-EDTA. Chromatin was eluted in an elution buffer (1% SDS-0.1 M NaHCO_3_) and reverse cross-linked by adding 0.3 M NaCl at 65°C overnight. Eluted DNA was precipitated, treated with proteinase K at 45°C for 2h and purified with Qiagen Min-Elute PCR purification columns. Purified DNA was used as a template for qPCR amplification of indicated regions of KSHV genome using primers listed in Table B in S1 Text.

### Low-cell chromatin immunoprecipitation assay

We performed LowCell ChIP using the LowCell ChIP Kit (Diagenode Inc.). Briefly, 1 million cells were fixed as described above followed by a PBS wash and re-suspending them in the kit-provided chromatin-shearing buffer. Chromatin was sonicated to an average size of 500bp using the Bioruptor Pico (Diagendode Inc.) and chromatin with specific antibodies were precipitated as described above.

### ChIP-seq and data analysis

TRExBCBL1-RTA cells were used for ChIP-seq assay by precipitating the chromatin from both un-induced and doxycycline induced (12h or 24h) cells with indicated antibodies. DNA extracted from the chromatin bound to respective targets and their appropriate controls was used for preparing the DNA sequencing libraries using NEXTflex^TM^ Illumina ChIP-Seq Library Preparation Kit (Bioo Scientific Inc. TX, USA). Mature libraries were analyzed for purity and quantification using Bioanalyzer 2100 (Agilent Technologies, Inc.) and kappa library quantitation kit (Kappa Biosystems, Inc.), respectively. Sequencing of these libraries was done on Illumina NextSeq500 (Illumina Inc.) and the sequences were analyzed using CLC Workbench 10.0.1 (Qiagen Inc.). Briefly, the sequences obtained from input, control IgG antibody and specific antibody samples were mapped to the KSHV genome (Accession number GSE98058, https://www.ncbi.nlm.nih.gov/geo/query/acc.cgi?acc=GSE98058) using ‘map reads to the reference’ tool of the CLC Workbench 10.0.1. Mapped reads were analyzed for enriched peaks in ChIP samples, represented as peak score, with respect to input reads using the ‘ChIP-seq’ tool of the CLC Workbench 10.0.1 with the minimum peak-calling P-value set to 0.05 [53].

### PRMT5 knockdown

SmartPool siRNA for control or PRMT5 (Dharmacon, GE Life Sciences) was transfected into iSLK.219 cells using RNAiMax transfection procedure (Lipofectamine, Thermofisher Inc.). Lipofectamine RNAiMax reagent was diluted in Opti-MEM medium and combined with siRNA diluted in Opti-MEM medium and incubated 5 minutes at room temperature. siRNA-lipid complex was added to cells at a 30pmol final amount of siRNA per well of 6-well plate and incubated 96 h before harvesting.

pGIPz shPRMT5 and pGIPzshControl (Dharmacon, GE Life Sciences) vectors were transfected with packaging plasmids into 293Lenti-X T cells (Clontech Laboratories, Inc.) and induced with NaB for 12 hours. Supernatant was collected every 12 hours over the next 3 days and lentiviral particles were concentrated by centrifugation. Lentivirus was added to BCBL-1 cells treated with polybrene and the transduction efficiencies were monitored by the visualization of GFP. Transduced cells were selected with 1μg/mL puromycin and the knockdown efficiencies of both methods were tested with Western blotting and comparative qPCR. qPCR analysis was performed by calculating the relative fold change values against the control knock-down samples and then the fold change values from 3 replicates were averaged and displayed in the graphs Fig 4D and 4E with standard deviation represented by error bars.

### KSHV transcriptome analysis

293L with BAC36WT, BAC36Δ50, BAC36Δ59, BAC16WT, or BAC16RTASTOP were transiently transfected with pLVXdsRedORF59-Flag, pLVX-RTA, or pLVXdsRed, vector control using PEI transfection reagent and cells were harvested 36 hours post transfection. Additionally, above cell lines were lentivirally transduced with either pLVXdsRedORF59-Flag or vector control and the expression was confirmed by Western blot analysis. PRMT5 knock-down cell samples (detailed above) were also subjected to transcriptome analysis by real-time qPCR analysis. Briefly, approximately 2 million cells were harvested and RNA was isolated using GE RNA Spin Kit, samples were eluted in 40μ RNAse-free water. cDNA was generated using High-Capacity RNA-to-cDNA kit (Applied Biosystems Inc.) according to manufacturer’s instructions. qPCR was performed using cDNA as template for amplifying viral ORF targets listed in Table A in S1 Text. Target genes were quantitatively assessed by comparative CT values and normalized with untreated/control samples. Fold changes were calculated using ΔΔCt method and the error bars represent standard deviation of three experiment replicates.

### *In vitro* translation for [^35^S]-methionine labeled proteins, *in vitro* binding assay and autoradiography

GST proteins were purified as previously described[50]. Briefly, induced BL-21 bacterial culture with indicated GST-fusion plasmids were harvested and the purified proteins were collected on Glutathione beads. Aliqots were taken and resolved by SDS-PAGE and coomassie staining to estimate relative quantity.

TNT-T7 Quick Coupled Transcription/Translation System (Promega Inc.) was used to generate ^35^S-methionine labeled proteins according to manufacturer’s instructions. Briefly, reaction components were thawed on ice and combined with plasmid DNA template (5μg) and 2μl [^35^S]-methionine followed by incubation for 3 h at 30°C. Expression was confirmed by resolving small aliquots of translated protein on a SDS-PAGE and exposing to autoradiography screens.

*In vitro* translated [^35^S] methionine labeled proteins were first pre-cleared by rotating at 4°C for 30 minutes with Control-GST protein beads. Pre-cleared samples were then combined with equal amounts of respective GST-fusion proteins and the final volume of the binding mixture was brought up to 700 μl with *in vitro* binding buffer (1XPBS, 10% glycerol, 0.1%NP-40) supplemented with 1μM DTT and protease inhibitors. Samples were rotated 4°C overnight then washed 3 times with binding buffer before being resuspended in 1X PAGE Buffer. All samples, including inputs were then incubated at 95°C for 5 minutes before being resolved by SDS-PAGE and imaged by autoradiography.

### Accession numbers

The next generation sequence data of ChIP-seq are deposited to NCBI genbank with accession number Series GSE98058, that includes subseries GSE98057, GSE98087, GSE100044.

### Statistical analysis

All the data were analyzed using Prism 6 software (Graphpad Inc.) for significance.

## Supporting information

S1 Text (DOCX)
