# Supplementary material for: KSHV encoded ORF59 modulates histone arginine methylation of the viral genome to promote viral reactivation
Source: PLoS Pathog. 2017 Jul 5;13(7):e1006482. doi: 10.1371/journal.ppat.1006482 (PMC5513536; doi:10.1371/journal.ppat.1006482)
Supplement: S1 Text — (DOCX) [file ppat.1006482.s001.docx]

**S1 Text**

**Supplemental Figures:**

ORF59 was cloned into a lentiviral vector, pLVxEGFP-C1-Flag to obtain a GFP-fused ORF59-Flag. pLVxEGFP-C1-ORF59-Flag (GFP-59-Flag) and the control PLVxEGFP-C1-Flag (GFP-Flag) were transduced into 293L cells. GFP fused ORF59 showed tight nuclear localization as compared to the GFP signal (Fig. A *part A*) Expression of GFP and flag tagged ORF59 was confirmed by the detection of flag-tagged protein in the lysate from these stable cells (Fig. A *part B*). To identify the proteins binding to ORF59, these stable cells were subjected for immunoprecipitaiton with anti-Flag antibody and the bound complexes were resolved on a SDS-PAGE. The gel was coomassie stained and the unique specific bands in the GFP-ORF59-Flag samples (Fig. A, *part C*) were excised for protein sequencing. GFP-Flag and GFP-ORF59-Flag are indicated with red asterisks (Fig. A, *part C*). One unique binding partner was PRMT5 (protein arginine methyltransferase 5), a type II methyltransferase responsible for adding two methyl groups to specific arginines in a symmetric fashion.

Arginine residues on histone tails are modified in a mono, or di-methylated fashion. Di-methylated residues are chemically identical; however, the dimethylation can be in either a symmetric, or asymmetric conformation. This is significant because it arises as a result of the structural and steric differences and has contrasting effects on chromatin landscape and the regulation of gene transcription. For example, the presence of *symmetric* dimethylation on H4R3 residues is on a transcriptionally “repressive” mark while the *asymmetric* dimethylation of that same residue is an “activating” mark.

**Figure A:** ORF59 associates with PRMT5 independent of any other viral proteins. **A.** 293L cells were transduced with either pLVx-EGFP-C1-Flag or pLVX-ORF59-GFP-Flag. **B.** Expression of GFP-Flag (lane 1) and GFP-ORF59-Flag (lane 2) were confirmed by anti-Flag immunoblot. M-protein marker. **C.** Unique protein bands in lane 2 were excised and proteins were sequenced by MALDI-TOF analysis. Red stars indicate GFP-Flag (lane 1) or ORF59-GFP-Flag (lane2).


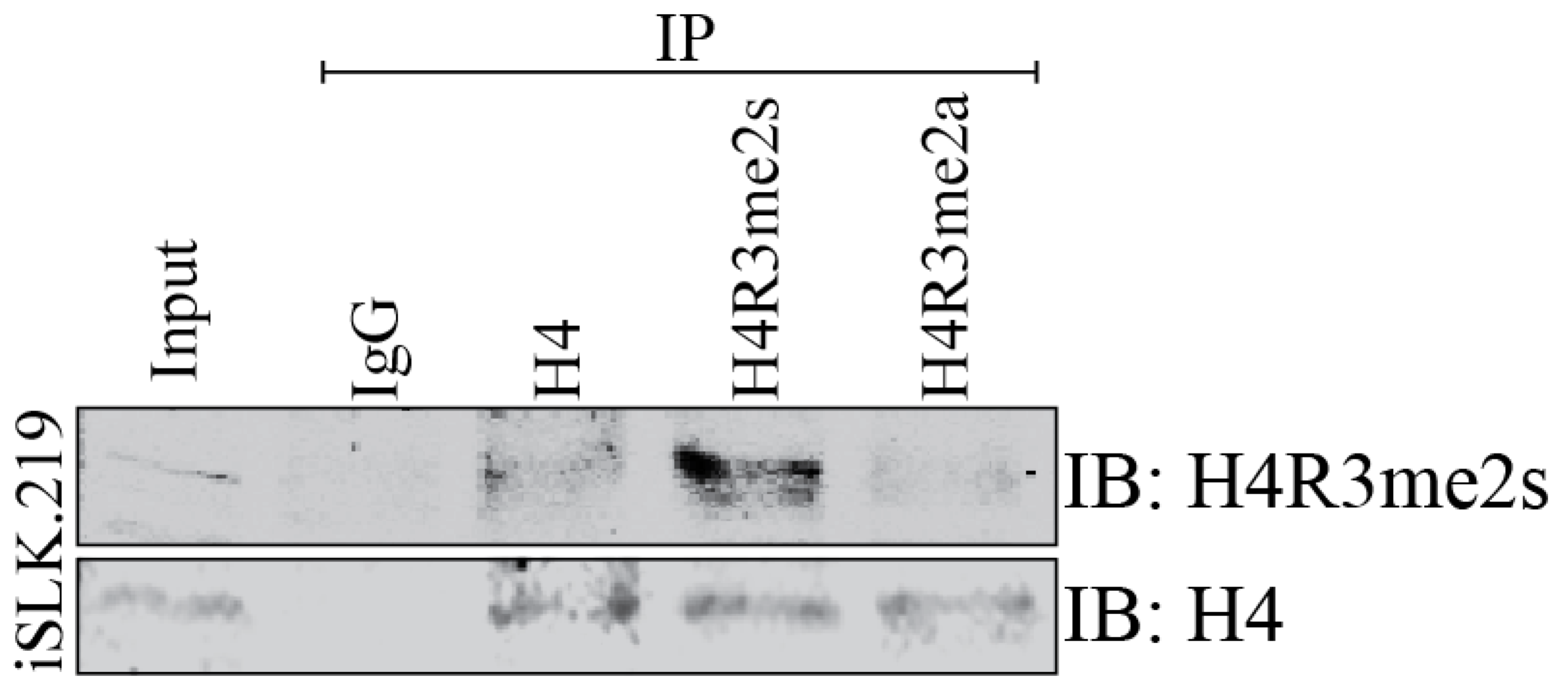
 We identified and confirmed an interaction between ORF59 and PRMT5, a type II arginine methyltransferase that bestows methyl groups in a symmetric conformation. We hypothesized that the interaction of ORF59 with MRPT5 may affect the levels of H4R3me2s on the viral chromatin, therefore wanted to determine the levels of symmetrically methylated H4R3 on latent and lytically reactivated cells (expressing ORF59) by ChIP-seq. However, due to the similar chemical nature of symmetric and asymmetric marks, we wanted to verify the specificity of the antibodies used to perform the ChIP for Next-Generation Sequencing (NGS). iSLK.219 cells were lysed and subjected for immunoprecipitation with total histone H4, H4R3me2s, and H4R3me2a. As expected, H4R3me2s antibody immunoprecipitated H4R3me2s histones most strongly, but did *not* have an affinity for the asymmetrically modified residues (Fig. B). This enhanced our confidence that the ChIP Seq data shown in Figure 4 represents the levels of H4R3me2s, the repressive mark.

**Figure B:** H4R3me2s antibody is specific for symmetrically methylated H4R3 but not the asymmetrically modified H4R3. IPs with indicated antibodies were performed on iSLK.219 cells followed by detection with H4R3me2s antibody, which specifically detected the symmetrically methylated form of H4R3.

In order to determine if ORF59 was enriched along the viral genome in the absence of viral replication, we performed the ORF59 ChIP Seq on iSLKBac16WT cells induced for lytic reactivation by the addition of doxycycline for 24h as well as the same cells treated with 0.5mM PFA. ChIP Seq analysis was performed as described in Materials and Methods. Briefly, the reads were mapped to the KSHV genome using the “map reads to reference” tool of the CLC Genomics Workbench 10.0.1 and ChIP Peaks were identified using the “ChIP Seq” tool and a minimum P-value for peak calling of 0.05. ORF59 shows significant binding to the viral genome upon reactivation, even in the absence of lytic replication (Fig. C *parts A and B*) Accession number: GSE98058.


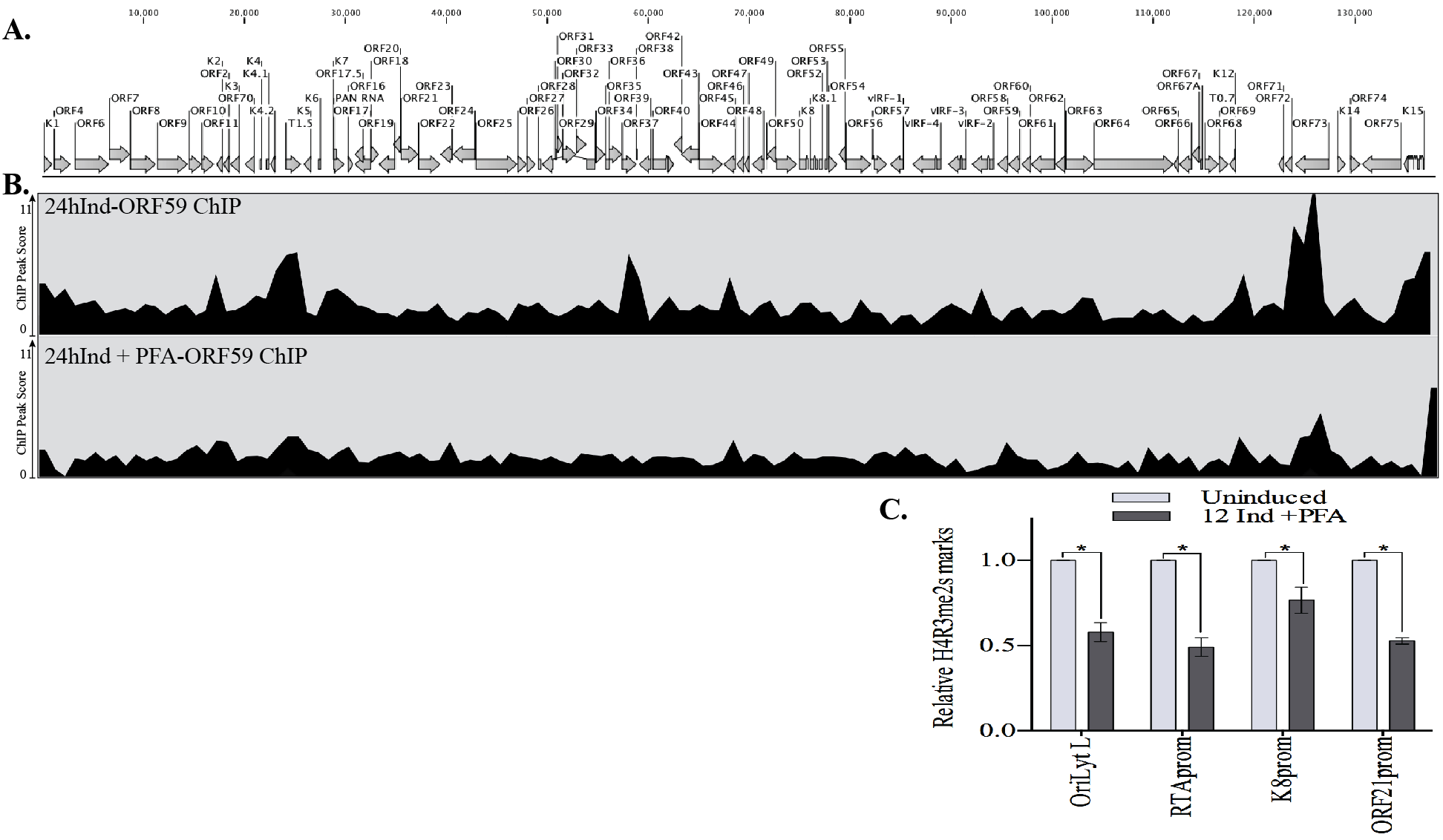
Furthermore, we tested the levels of H4R3me2s on TRExBCBL1-RTA cells treated with 0.5mM PFA and induced for lytic reactivation for 12h and assessed H4R3me2s enrichment by qPCR at specific viral promoter regions. Compared to uninduced cells, these PFA-treated induced TRExBCBL1-RTA cells still showed a significant reduction in H4R3me2s (Fig. C *part C*).

**Figure C:** ORF59 bound to viral chromatin even in the presence of replication inhibitor, PFA and reduced symmetric di-methylation of H4R3me2.**A.** Schematic of the KSHV genome. **B.** iSLKBac16WT cells were induced for 24h by addition of doxycycline and either untreated (above panel) or treated with 0.5mM PFA (below panel). Cells were harvested and fixed, chromatins were sheared and immunoprecipitated with specific anti-ORF59 antibody. ChIP DNA was then used to prepare libraries that were sequenced on Illumina NextSeq500 platform. ORF59 is enriched at various viral loci irrespective of genome replication. **C.** TRExBCBL1-RTA cells uninduced or 12h induced and treated with 0.5mM PFA were harvested and fixed, chromatins were sheared and immunoprecipitated with specific anti-H4R3me2s antibody. qPCR analysis revealed decrease in H4R3me2s marks even with PFA treatment.


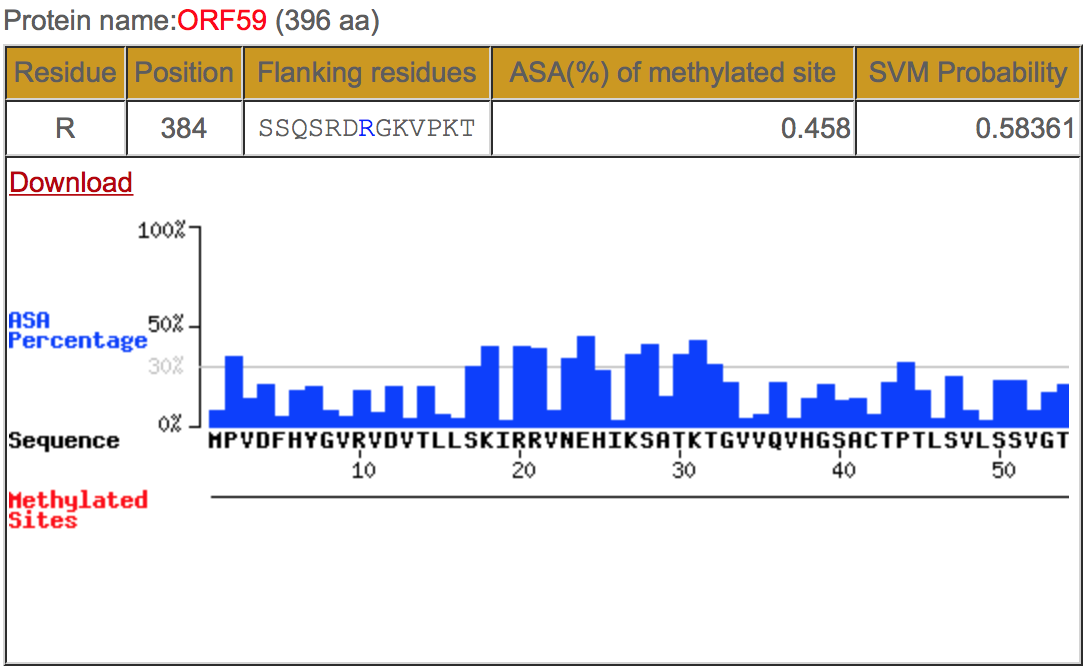


**Figure D:** Arginine methylation site of ORF59 identified by a methylation prediction software, MASA (Methylation site based on the Accessible Surface Area) available at [http://masa.mbc.nctu.edu.tw/predict.php](https://na01.safelinks.protection.outlook.com/?url=http%3A%2F%2Fmasa.mbc.nctu.edu.tw%2Fpredict.php&data=01%7C01%7Cscverma%40med.unr.edu%7Cc03c77b8c1e94513eb8708d48bfc63b0%7C523b4bfc0ebd4c03b2b96f6a17fd31d8%7C1&sdata=IhqBEvzepLV3bzZ0x0qpIY4jgpNHPzAghBBLdf0Umx4%3D&reserved=0).

**Table A: KSHV ORF-Scanning Primers:**

| **Name sense** |  | **Primer Sequence:Sense** |  | **Name anti-sense** |  | **Primer Sequence:Antisense** |  |
| --- | --- | --- | --- | --- | --- | --- | --- |
| S-K1 | 5'- | CCAAATTTGTGCCCTGGAGTGA | -3' | AS-K1 | 5'- | GCCCAGATTGTTCCACACAAGT | -3' |
| S-ORF4 | 5'- | AAGCCTGAAGACACCCATACAGCA | -3' | AS-ORF4 | 5'- | TGTGTATTTGGCTGGGATGTAGT | -3' |
| S-ORF6 | 5'- | TTCGAGGCAGAAGCGGTTAAGA | -3' | AS-ORF6 | 5'- | TAAGGGCCCAGGTAGTAAATCACG | -3' |
| S-ORF7 | 5'- | AAACGCACACGAATGCTCAGGT | -3' | AS-ORF7 | 5'- | GCTCCGGTGCTTGTTGCATGTTAT | -3' |
| S-ORF8 | 5'- | ACGGTCCAGTACTTCCACACAA | -3' | AS-ORF8 | 5'- | TCTCTTTCTGCGGCTGGCA | -3' |
| S-ORF9 | 5'- | ACAAGCTGGACACAGTGGCTAA | -3' | AS-ORF9 | 5'- | AGATCCATAACCAGGACCGAGT | -3' |
| S-ORF10 | 5'- | ACACAATAGTGGGCGTGGCAAT | -3' | AS-ORF10 | 5'- | TACGTGGCAAGGTGGTGTACAGA | -3' |
| S-ORF11 | 5'- | TTTACATTCGACAACACGCACCGC | -3' | AS-ORF11 | 5'- | ACGGCGATGTTCGCAGAGA | -3' |
| S-K2 | 5'- | AAGCTCGCCGATGGCTTT | -3' | AS-K2 | 5'- | ATTTGGGTGGACTGTAGTGCGT | -3' |
| S-ORF2 | 5'- | TTGCGGTTGATACCAAACTCGGGA | -3' | AS-ORF2 | 5'- | AATCGAAAGCCATGTGCGCC | -3' |
| S-K3 | 5'- | TGTGCATCGCGACTCAGAAGAA | -3' | AS-K3 | 5'- | GCACCAACATAACCGCCACAGTAA | -3' |
| S-ORF70 | 5'- | ACATTGCCAGCTATTCCCTCT | -3' | AS-ORF70 | 5'- | TCCATGGAAGAAACAGACCGGA | -3' |
| S-K4 | 5'- | TGTGCTGACTGCCTTGCTTTGT | -3' | AS-K4 | 5'- | AAATATCACACCCGGCTTGCTG | -3' |
| S-K4.1 | 5'- | TAACGGCCCGTGTGTGTTGAGT | -3' | AS-K4.1 | 5'- | GCGGTTACTAACGACGGTTACA | -3' |
| S-K4.2 | 5'- | CTAGTGAGCGTCTTGTTGCTGT | -3' | AS-K4.2 | 5'- | ACGATAAAGACCAGCGTTGCCA | -3' |
| S-K5 | 5'- | AAGCACTTGGCTAACAGTGTCTCG | -3' | AS-K5 | 5'- | CACCACCACAGACATCAGCAAA | -3' |
| S-K6 | 5'- | AAGATATCACTGCGGCCCAACT | -3' | AS-K6 | 5'- | TTCTGTGGTGCGCTTCTGGGAAA | -3' |
| S-K7 | 5'- | CGTTGTTTCGGTTCTGTGTTTGTC | -3' | AS-K7 | 5'- | ACACACCATTACAGCACTAGCC | -3' |
| S-ORF16 | 5'- | GGTTAGCGGCGATGGAAACATGAA | -3' | AS-ORF16 | 5'- | GGGTCCGATTGCTTCATACGC | -3' |
| S-ORF17 | 5'- | TCCGGATCAGGTGACGGATTAT | -3' | AS-ORF17 | 5'- | TAGGAAGGCTGGCGACGTGAT | -3' |
| S-ORF17.5 | 5'- | CACCATACGGCTACGCTTACAA | -3' | AS-ORF17.5 | 5'- | AAGATATCCTTGTGATACGCCGGCTC | -3' |
| S-ORF18 | 5'- | ACAGCCTCTGGATGCACTTGAA | -3' | AS-ORF18 | 5'- | GGGAGCACACTTCCCAGATTAAACA | -3' |
| S-ORF19 | 5'- | CCACAAACAACCCGGACCATTT | -3' | AS-ORF19 | 5'- | ACGTCCAGGCCCTCTTCTGTT | -3' |
| S-ORF20 | 5'- | AAGATGGACAGAGGCGGGAAGATT | -3' | AS-ORF20 | 5'- | GCAGGTGTGCCTTTGTCTTTCGTT | -3' |
| S-ORF21 | 5'- | ACGACGAATCAAGCACCTCCACAA | -3' | AS-ORF21 | 5'- | CGTAGAGGGAGTTGTCGTGCATTA | -3' |
| S-ORF22 | 5'- | TACCCAATGAATGTCATGGCCG | -3' | AS-ORF22 | 5'- | AATCCGGGAGACCGAACAGAAGAT | -3' |
| S-ORF23 | 5'- | CCAAAGACCGTCAAAGCGCC | -3' | AS-ORF23 | 5'- | TACTTGGCAAGAAATCCGAGCACC | -3' |
| S-ORF24 | 5'- | TGCCATCGCTCACAACGTAGA | -3' | AS-ORF24 | 5'- | GGGTCCTTGCGTTGGATTT | -3' |
| S-ORF25 | 5'- | TCGAAGCAATGATTCACGGACAGG | -3' | AS-ORF25 | 5'- | CCTCCTTAGGGATGCTTCCATTC | -3' |
| S-ORF26 | 5'- | CCCGGATGATGTAAATATGGCGGA | -3' | AS-ORF26 | 5'- | TGGGAACCAAGGCTGATAGGAT | -3' |
| S-ORF27 | 5'- | GGCGTCATCTGATATTCTGTCGGT | -3' | AS-ORF27 | 5'- | CACGATGAAGCTTTCGAGCCAT | -3' |
| S-ORF28 | 5'- | AAGCCTCCCGTGATTGGTCTTA | -3' | AS-ORF28 | 5'- | AGGGTGCGAAGGACCTGATA | -3' |
| S-ORF29 | 5'- | ATCCCGTGATATGCGTCCCTGT | -3' | AS-ORF29 | 5'- | GCCGAATGCGTTCTCCTGCATA | -3' |
| S-ORF30 | 5'- | TGGATCCTGGACATGTGGTGAA | -3' | AS-ORF30 | 5'- | TCCATCAGCAGGCAGAGTCTTT | -3' |
| S-ORF31 | 5'- | ATGTCAGTTCCACGGCGTGTTT | -3' | AS-ORF31 | 5'- | TGAATGTTGCCGAGCATGCAGT | -3' |
| S-ORF32 | 5'- | ATGTGTGTGTTTACGAACGCGG | -3' | AS-ORF32 | 5'- | TGTCCACAAAGTCGCACTGCTT | -3' |
| S-ORF33 | 5'- | AAGGTCTTTAACGCCTGCACCT | -3' | AS-ORF33 | 5'- | TCCGTTAATTCCCACAGCCACA | -3' |
| S-ORF34 | 5'- | TGGACGTGGGCTTTGACTCTGAAT | -3' | AS-ORF34 | 5'- | AACCGCAGTGCTGCTTGTA | -3' |
| S-ORF35 | 5'- | ATCAACAGGAGGGCAGCTGTAT | -3' | AS-ORF35 | 5'- | TTTCAGCGCCTCAAACCTCTGG | -3' |
| S-ORF36 | 5'- | ACATGTCCTCGTTGGGCTACACT | -3' | AS-ORF36 | 5'- | TCATCACCACCCTGGGAATCAT | -3' |
| S-ORF37 | 5'- | TGGGCGAGTTTATTGGTAGTGAGG | -3' | AS-ORF37 | 5'- | TCTCCACTAGACAGCAGATGTGG | -3' |
| S-ORF38 | 5'- | CTATCTGCAAACGTCCCTCACA | -3' | AS-ORF38 | 5'- | GATTGCTCAAGCAACATGCCCT | -3' |
| S-ORF39 | 5'- | TATCGTGGACGGCATTGCCATAGT | -3' | AS-ORF39 | 5'- | CCTTAAGATGGCGACATAAAGCACGC | -3' |
| S-ORF40 | 5'- | CCGCATTATTGTCTGGCAGGTT | -3' | AS-ORF40 | 5'- | AGGTCACGGGCGTGTTCAGTAATA | -3' |
| S-ORF42 | 5'- | TCTGCTGCCTGTTATCGATGCT | -3' | AS-ORF42 | 5'- | TGGCAGTCAACGTCTCTATACCGT | -3' |
| S-ORF43 | 5'- | TGGGCGGGTGATCTTTGACA | -3' | AS-ORF43 | 5'- | TCCAGGTGGGTCATGATGTCTT | -3' |
| S-ORF44 | 5'- | TTTGGTGACGAGTTCCTCCACT | -3' | AS-ORF44 | 5'- | TGGGAGGAACGTATATCCCTGG | -3' |
| S-ORF45 | 5'- | GCTGCCAGAGACAACCAAACTT | -3' | AS-ORF45 | 5'- | AAGTACTGGGAGATTGGGTTGGGA | -3' |
| S-ORF46 | 5'- | GTGGATTTCCTTTCTGAGCCTCTC | -3' | AS-ORF46 | 5'- | TCCTGGCCTAAGATCACCACTT | -3' |
| S-ORF47 | 5'- | ACCATGTTGCGCAATTCAGGCA | -3' | AS-ORF47 | 5'- | CATTCACGCATGTTTCCACGGT | -3' |
| S-ORF48 | 5'- | TCCCACGGGAGACACATTCTTT | -3' | AS-ORF48 | 5'- | TGAAATCAAGCTAAGCTCCCGC | -3' |
| S-ORF50 | 5'- | AGGCATCCCAAGGCATTATTCGGA | -3' | AS-ORF50 | 5'- | CCCAGGCATTTGGCCTTCATTT | -3' |
| S-ORF49 | 5'- | AGGAGTTAGATACCCTGTCCGT | -3' | AS-ORF49 | 5'- | ACTGCTGCAGGGAATAAGCCAA | -3' |
| S-ORFK8 | 5'- | TGCCCAGAATGAAGGACATACC | -3' | AS-ORFK8 | 5'- | AACAGCGAGCTTGTCCAAGAGA | -3' |
| S-K8.1 | 5'- | AAATCCCTGTGGCGCTCCTAAT | -3' | AS-K8.1 | 5'- | TCGTAGGAACAGTTCATCCTGCCT | -3' |
| S-ORF52 | 5'- | TGGAGAATCGGGAGCTTCGGAAA | -3' | AS-ORF52 | 5'- | ATTGTCCTGACCCTGGCTTCGATT | -3' |
| S-ORF53 | 5'- | TGTCGCATCATCTACAGAGCGT | -3' | AS-ORF53 | 5'- | ATCAAGGCCCAGACGCTAGAAA | -3' |
| S-ORF54 | 5'- | AGCCGCATATGCCAGATTGTGT | -3' | AS-ORF54 | 5'- | GGGTTGTCTTCGTGGGTATGTT | -3' |
| S-ORF55 | 5'- | TGCGCTCCAGTCCCTCTTAAACAA | -3' | AS-ORF55 | 5'- | AGGCGTGAGAGGCAATACAGAA | -3' |
| S-ORF56 | 5'- | TGGTCCACAGATTCCCGTCAATAC | -3' | AS-ORF56 | 5'- | GGTGACAGACTAGTATCTTCAGTAGGCG | -3' |
| S-ORF57 | 5'- | GTCTGCAATGCGTTTGTTACCAG | -3' | AS-ORF57 | 5'- | TGACCTCGCCAAGAAGGTTACA | -3' |
| S-vIRF-1 | 5'- | ACCCGAATACATTTCTGGTGGG | -3' | AS-vIRF-1 | 5'- | ACTGCACCTCGCCGCAAA | -3' |
| S-vIRF-4 | 5'- | CCTTGGCCACGGATTCCCAATA | -3' | AS-vIRF-4 | 5'- | TTGAACAGCTGCTGCGGAACT | -3' |
| S-vIRF-3 | 5'- | CTGCATGTGCATTGATGTACCAC | -3' | AS-vIRF-3 | 5'- | ATTGAGCCCTCTGTTACCACGC | -3' |
| S-vIRF-2 | 5'- | TTCCACCGTTGTGTCGTGATGA | -3' | AS-vIRF-2 | 5'- | CAAGCTTGTCACCCACTTTGT | -3' |
| S-ORF58 | 5'- | TGTTTGTGACCTTACTGCTGGC | -3' | AS-ORF58 | 5'- | AGGAGAACAAATAGCGCTCGTC | -3' |
| S-ORF59 | 5'- | TGTGTAAAGTCCCGGGTTGGTT | -3' | AS-ORF59 | 5'- | TCCGGTATAGAATCGGGAACCT | -3' |
| S-ORF60 | 5'- | TATCAGTTTGTGCAGGGTGCGA | -3' | AS-ORF60 | 5'- | CCGAATCTCAATATTCACTGCCTCCC | -3' |
| S-ORF61 | 5'- | TCGATAACGAATACGAGCGCCT | -3' | AS-ORF61 | 5'- | TTAATGGCCATGCCCTGAGTGT | -3' |
| S-ORF62 | 5'- | AAGCGCCACAGACTCACAAAGT | -3' | AS-ORF62 | 5'- | TGTGGACACTCTGGACCATTGGA | -3' |
| S-ORF63 | 5'- | TGGAATTTCAACTCCGAGGACTGC | -3' | AS-ORF63 | 5'- | AAATACGCCGCGCGAACCAGT | -3' |
| S-ORF64 | 5'- | AGCACGGCCTGACAGAGAG | -3' | AS-ORF64 | 5'- | ACACATCATCGTCGTCAGAGGGT | -3' |
| S-ORF65 | 5'- | TGGATCATGACTACGCTCACCA | -3' | AS-ORF65 | 5'- | CCATCCTCCTCAGATAGGCCTCATAA | -3' |
| S-ORF66 | 5'- | TTCCTCCCACCAACGTGTTT | -3' | AS-ORF66 | 5'- | TGATTACGACGCGTGCTAGGGT | -3' |
| S-ORF67 | 5'- | TTCTCAAAGAACCTGGGCGTGT | -3' | AS-ORF67 | 5'- | AAACCGGCACCACCCTGT | -3' |
| S-ORF67A | 5'- | AGTACGCGTCTGACCAGCTTCT | -3' | AS-ORF67A | 5'- | AACACCAGAGTATGATCGCAGGCT | -3' |
| S-ORF68 | 5'- | ATTCACCAGTCGCTCCAACCAT | -3' | AS-ORF68 | 5'- | ATGCATGATCTCGCACAGGTCT | -3' |
| S-ORF69 | 5'- | ATCAGTGTCCAGCCACATCAGCTT | -3' | AS-ORF69 | 5'- | AACTTGCGAAGGCCGTTTCTGT | -3' |
| S-K12 | 5'- | TGCAACTCGTGTCCTGAATGCT | -3' | AS-K12 | 5'- | AGGCTTAACGGTGTTTGTGGCA | -3' |
| S-ORF71 | 5'- | TCGCAGCACACCACAGACATTCTT | -3' | AS-ORF71 | 5'- | AGGCCCATTAGGGTTTGCACTT | -3' |
| S-ORF72 | 5'- | AGCTCAGAAGCCTCACGCCTATTT | -3' | AS-ORF72 | 5'- | AAGTGACGTCCGTCGCTAAGACT | -3' |
| S-K14 | 5'- | TCATTACCTTGGGTGGCGTTCA | -3' | AS-K14 | 5'- | TATGAATGGCTGTACGTGGCGA | -3' |
| S-ORF74 | 5'- | AAACATGACTGCAGACTGGCGA | -3' | AS-ORF74 | 5'- | ACAGCAGCACCACAGCAACAAT | -3' |
| S-ORF75 | 5'- | TTGGCTACGGGCCAAACATAAACC | -3' | AS-ORF75 | 5'- | AAACTGAGGTGGGTGTGGTTCT | -3' |
| S-K15 | 5'- | TTTAGTGTCCGGTGCGGAAGAA | -3' | AS-K15 | 5'- | AACCTCCTCATACAGGTCGTCT | -3' |
| S-ORF73 | 5'- | TTGCCTATACCAGGAAGTCCCACA | -3' | AS-ORF73 | 5'- | GGAGGAAGACGTGGTTACGGG | -3' |
| ST-P53 | 5'- | TGAGACTGGGTCTCGCTTTGTT | -3' | AS-TP53 | 5'- | AAAGTTGGCTGGCCATGGTG | -3' |
| S-PRB | 5'- | CCGGCTAAATACACTTTGTGAACGCC | -3' | AS-PRB | 5'- | GCATATGCCATACATGGAACACA | -3' |
| S-IRF3 | 5'- | ATGGCCAGTCACACTGCCAGA | -3' | AS-IRF3 | 5'- | TCGCTCACTGCCCAGTATGTGT | -3' |
| S-IRF7 | 5'- | ACCAGAAGCAGCTGCGCTACA | -3' | AS-IRF7 | 5'- | TGTCACAGTTCCGAGGCAGCA | -3' |
| S-1.4kb | 5'- | GCTTCCAGCGAACGGAAATAAG | -3' | AS-1.4kb | 5'- | ACGGAGAATAGACCAGCCTGAA | -3’ |
| S-PAN RNA | 5'- | GGCTAGTGCTGTAATGGTGTGT | -3' | AS-PAN RNA | 5'- | AACATTGAAAGAGCGCTCCCAG | -3’ |
| S-ACTB | 5'- | ACAATGTGGCCGAGGACTTTGA | -3' | AS-ACTB | 5'- | TGTGTGGACTTGGGAGAGGACT | -3' |
| S-GAPDH | 5'- | CAGCAAGAGCACAAGAGGAAGA | -3' | AS-GAPDH | 5'- | TTGATGGTACATGACAAGGTGCGG | -3' |
| S-18S | 5'- | ACCAGAGCGAAAGCATTTGCCA | -3' | AS-18S | 5'- | AGACTTTGGTTTCCCGGAAGCTG | -3' |

Interestingly, we determined that similarly to other histone modifications, the enrichment of the suppressive H4R3me2s mark on the KSHV genome was lesser at several promoters upon lytic reactivation (Fig. 4). PRMT5 is the main enzyme responsible for bestowing this epigenetic mark and after discovering that the knockdown of PRMT5 facilitates lytic gene transcription, we wanted to test the levels of H4R3me2s in the PRMT5 knockdown cells. Although the removal of methyl groups on arginine residues of histones was not well understood, we found that in KSHV positive cell lines with reduced expression of PRMT5, had reduced levels of H4R3me2s marks at various viral promoter regions. The promoter regions tested were chosen based on the ChIP-Seq results showing the greatest changes between latent and lytic levels of H4R3me2s marks and the primers for those regions are listed below in the table.

**Table B: qPCR Analysis Primers: KSHV Gene Promoter Regions:**

| **Primer** | **Primer Sequence (Sense, Anti-sense)** |
| --- | --- |
| OriLyt-L | GCTTCCAGCGAACGGAAATAAG |
|  | ACGGAGAATAGACCAGCCTGAA |
| K5prom | TCGCAAGCACGCGCATATAA |
|  | TACGACACGCCTTGTGTACAGT |
| ORF21prom | AGCAGTGAGAAAGGCGCACAAT |
|  | TCCGACTCGATCCATTACATCGCT |
| ORF34prom | TTTGCTTTGAGCTCGCTCGTGT |
|  | GGCATCACGTTGGAAACCAAGA |
| ORF35prom | ATCAACAGGAGGGCAGCTGTAT |
|  | TTTCAGCGCCTCAAACCTCTGG |
| RTAprom | ATGTCAGATGCTGTTGCCTGG |
|  | AGCGTACATGTGACTGAACATGG |
| K8prom | CGGATATACCGTCACCTTCTGGT |
|  | TGCGAACACTTCAGTCTCGGAA |
| vIRF1 region | GCTGGTAACGGTGAATTTGCCA |
|  | GGCATTCTGCTGACTAGCTCTT |
| ORF63 | GCTACACATACTCATGCACCCA |
|  | CGCAGCTTAGCAGCCTGTATTT |
| ORF64prom | ACAGTTTACAGACGCTCAGGCT |
|  | TTCGACAAACAGTTTCCGGAGG |
| Genome Region 64-65 | TGCAGATGATCCCGCCTTTGAA |
|  | TGGATCATGACTACGCTCACCA |
| OriLyt-R | TTAGCCACCCATTTCCCGGGG |
|  | CTGGGTCTGTTCGGTAGATGG |
| LANAprom | ATCCGGGCGTGAGAAACAGAAA |
|  | TATAAACAGTGAGTAGCGCCCG |

To better understand the nature of interaction between ORF59, PRMT5 and COPR5, we systematically cloned truncated regions of PRMT5 into a Flag-tagged vector using primers listed below.

**Table C: Cloning Primers for PRMT5, COPR5 truncated mutants:**

| **Primer** | **Primer Sequence** |
| --- | --- |
| S-COPR5BamHI-pa3F | ctcggatccgccatgGACCTTCAGGCCGCCGGG |
| AS-COPR5EcoRI-pa3F | gcagaattccATCTTCAGCATCGTCAAACTG |
| AS-COPR5:1-140aa pLVxBamHI | tttGGATCCCTCTTGAGAAATGCTC |
| S-COPR5:141-184aa pLVxEcoRI | tttGAATTCACTTAAACCTTGGGTGTG |
| S-PRMT5-BamHI-pa3F | gtaccgagctcggatccATGGCGGCGATGGCGGTCGGG |
| AS-PRMT5-EcoRI-pa3F | ggatatctgcagaattcTGAGGCCAATGGTATATGAGCG |
| AS-PRMT51-210aa-EcoRI-pa3F | ggatatctgcagaattcTAGCCCCAATTTCAAGAGCCAC |
| S-PRMT5210-420aa-BamHI-pa3F | gtaccgagctcggatccATGGACCTCCCATCTAATCAT |
| AS-PRMT5210-420aa-EcoRI-pa3F | ggatatctgcagaattcTCATGTCTGATGAGACTACGGT |
| S-PRMT5420-637aa-BamHI-pa3F | gtaccgagctcggatccATGAGGGAATGGGTGGCTCCA |
